# ECDEP: identifying essential proteins based on evolutionary community discovery and subcellular localization

**DOI:** 10.1186/s12864-024-10019-5

**Published:** 2024-01-26

**Authors:** Chen Ye, Qi Wu, Shuxia Chen, Xuemei Zhang, Wenwen Xu, Yunzhi Wu, Youhua Zhang, Yi Yue

**Affiliations:** 1 School of Information and Artificial Intelligence, Anhui Agricultural University, Hefei, Anhui 230036 China; 2 Anhui Beidou Precision Agriculture Information Engineering Research Center, Anhui Agricultural University, Hefei, 230036 China

**Keywords:** Essential protein, Evolutionary community discovery, Protein–protein interaction network, Subcellular localization, Gene expression

## Abstract

**Background:**

In cellular activities, essential proteins play a vital role and are instrumental in comprehending fundamental biological necessities and identifying pathogenic genes. Current deep learning approaches for predicting essential proteins underutilize the potential of gene expression data and are inadequate for the exploration of dynamic networks with limited evaluation across diverse species.

**Results:**

We introduce ECDEP, an essential protein identification model based on evolutionary community discovery. ECDEP integrates temporal gene expression data with a protein–protein interaction (PPI) network and employs the 3-Sigma rule to eliminate outliers at each time point, constructing a dynamic network. Next, we utilize edge birth and death information to establish an interaction streaming source to feed into the evolutionary community discovery algorithm and then identify overlapping communities during the evolution of the dynamic network. SVM recursive feature elimination (RFE) is applied to extract the most informative communities, which are combined with subcellular localization data for classification predictions.

We assess the performance of ECDEP by comparing it against ten centrality methods, four shallow machine learning methods with RFE, and two deep learning methods that incorporate multiple biological data sources on *Saccharomyces. Cerevisiae (S. cerevisiae)*, *Homo sapiens (H. sapiens)*, *Mus musculus*, and *Caenorhabditis elegans*. ECDEP achieves an AP value of 0.86 on the *H. sapiens* dataset and the contribution ratio of community features in classification reaches 0.54 on the *S. cerevisiae* (Krogan) dataset.

**Conclusions:**

Our proposed method adeptly integrates network dynamics and yields outstanding results across various datasets. Furthermore, the incorporation of evolutionary community discovery algorithms amplifies the capacity of gene expression data in classification.

**Supplementary Information:**

The online version contains supplementary material available at 10.1186/s12864-024-10019-5.

## Introduction

In gene knockout and parallel analysis of genome function in *Saccharomyces cerevisiae* (*S. cerevisiae*), researchers have unveiled a phenomenon: the deletion of specific genes, commonly referred to as essential genes, can result in the demise or infertility of organisms. These essential genes give rise to essential proteins that oversee the fundamental requirements of life, exert a profound influence on cellular metabolism and differentiation [1, 2], facilitate the elucidation of cell growth and regulatory mechanisms, pinpoint pathogenic genes, and unveil potential drug targets [3, 4]. In the early stages of the research field, gene knockout, RNA interference, and transposon mutagenesis served as the foundational techniques for delving into gene functionality [5–7]. Despite their ability to yield precise sets of essential genes, these approaches required substantial resource allocation and time investments.

With the accumulation of high-throughput data and the completion of extensive proteome sequencing projects, technologies, including the *S. cerevisiae* two-hybrid system [8], affinity purification [9], and microarray analysis [10], have ushered in a wealth of protein interaction data. In the realm of post-genomic research, experiments have illuminated a compelling insight: the phenotypic outcomes resulting from gene deletions in *S. cerevisiae* are substantially affected by the topological positions of their protein products within the molecular interaction network [11]. This revelation has ignited an ongoing surge of methods centered around the network-based identification of essential proteins. As research advances, it has become evident that certain highly connected central nodes may not necessarily qualify as essential proteins. Consequently, the research focus has pivoted from the global topology of proteins to the local topology, exemplified by metrics such as the edge clustering coefficient [12]. This coefficient encapsulates the tightness of connections between nodes at both ends of an interacting edge and their direct surroundings, serving as a critical indicator of potential participation in network community structures. Local average connectivity (LAC) [13] and local interaction density (LID) [14] also evaluate protein essentiality by scrutinizing local neighborhood relationships. Subsequent research uncovered that high-throughput protein–protein interaction (PPI) networks present false positives. To address this challenge, researchers have embarked on the integration of diverse biological information and network structures. Notably, owing to the tendency of essential proteins to congregate into highly interconnected clusters, gene expression profiles have emerged as valuable data that researchers increasingly leverage [15–18].

In recent years, the development of multi-layer network scoring methodologies, grounded in a wealth of biological information sources, has emerged as a focal point in research. RWHN [19] approach constructs heterogeneous networks that interweave PPI networks and protein domain, then establishes a transition probability matrix based on normalization operation. Simultaneously, researchers often harness gene expression data to forge co-expression networks or weighted networks, employing iterative algorithms to gauge protein significance [20–22]. Another focusing area of research pertains to the dynamic attributes of networks. JDC [23], for instance, utilizes threshold calculations to binarize network fluctuations, subsequently combining degree centrality and the Jaccard similarity index to compute JDC scores. Meanwhile, CTF [24] identifies essential proteins through edge features and multi-source information fusion, culminating in edge-weighted PPI networks entwined with dynamic PPI data. The combination of gene expression data and its utilization to provide dynamism constitutes a widely adopted approach among researchers. Nevertheless, such dynamism is typically employed in the context of scoring methodologies, with scant exploration into the intricate relationship aggregations arising during the dynamic evolution process within deep learning methods.

Many machine learning and deep learning techniques have been deployed in the quest to predict essential proteins. Given the intricate nature of network topology and biological features, a lot of methods have emerged for the selection of pertinent features, often through the prism of feature engineering. Within this landscape, Support Vector Machines (SVM) [25] and ensemble learning methods [26] have emerged as conventional machine learning methods. On the other side, deep learning approaches are designed grounded in the inherent characteristics of biological data. For instance, DeepEP [27] melds PPI networks with gene expression data, leveraging node2vec technology [28] to extract both topological and semantic features from the PPI network, then treats gene expression data as images and subsequently extracts its features through convolutional neural networks. Meanwhile, Zeng et al. [29] introduced a method that harmonizes multiple biological information sources, deploying bidirectional long short-term memory (LSTM) networks to discern features from gene expression data, and then integrating PPI networks and subcellular localization data to enhance predictive capabilities. In a similar vein, MBIEP [30] capitalizes on depth-wise separable convolution to extract gene expression data features across diverse experimental contexts, while also processing subcellular localization data. While these methods each exhibit their unique strengths, it is worth noting that feature engineering-based approaches often necessitate the provision of an initial feature space. Moreover, the DeepEP model is bound by specific requirements concerning the input data structure of gene expression data. Additionally, models reliant on LSTM may encounter performance challenges due to the relatively short time course of gene expression data. The MBIEP model, while using one-dimensional convolution, faces limitations associated with input data requirements and a dearth of experimental projects in temporal and different environments, thereby constraining its applicability scope. Furthermore, the model's performance on *H. sapiens* datasets falls short of expectations.

Upon a summarization of prior research, it becomes evident that gene expression profiles, in isolation, contribute minimally to classification and remain underutilized. Furthermore, the exploration of dynamic networks within the realm of deep learning remains a relatively underdeveloped area. Existing deep learning methods often impose stringent data prerequisites, presenting a hurdle to their practical application. As a response to these challenges, we introduce the ECDEP model, founded upon an evolutionary community discovery algorithm. The central objective of ECDEP is to unlock more effective leverage of the dynamic attributes encapsulated within gene expression data. Within this framework, inspired by the principles expounded in the works of IFPA [31] and JDC [23], a dynamic PPI network is sculpted using the 3-sigma rule. Notably, gene expression data does not directly constitute the features governing classification decisions; rather, its primary role lies in the construction of dynamic networks. We then analogized the PPI as a social network, with the TILES [32] algorithm harnessed to unearth communities during the dynamic changes. Importantly, the TILES algorithm concerns network topology during the community discovery process, thus, we obviate the need for an extra feature extraction from the static PPI network to prevent redundancy. Through observation windows, we extract communities acquired at different observation intervals. The SVM recursive feature elimination (SVM-RFE) method is subsequently employed to pick 64 informative communities as sample features. Moreover, ECDEP incorporates subcellular localization as another feature, drawing inspiration from the processing method applied in MBIEP [30]. Proteins corresponding to subcellular localization are ranked in descending order, and the top n subcellular localization are selected. We exclude data from the 11th to the 64th positions, as they did not ameliorate model performance according to the conclusion from MBIEP. Ultimately, ECDEP utilizes fully connected layers to process the community features derived from dynamic networks, alongside features derived from subcellular localization. Once these features are condensed into one-dimensional vectors of uniform length, they are seamlessly integrated into the final classification prediction module.

We substantiate these propositions through extensive comparative experiments conducted across three *S. cerevisiae* databases, as well as three additional species: *Homo sapiens* (*H. sapiens*), *Mus musculus* (*M. musculus*), and *Caenorhabditis elegans* (*C. elegans*). Initially, the ECDEP model is compared with ten centrality methods, including degree centrality (DC) [11], betweenness centrality (BC) [33], closeness centrality (CC) [34], subgraph centrality (SC) [35], eigenvector centrality (EC) [36], maximum neighborhood component centrality (MNC) [37], local average connectivity (LAC) [13], local interaction density (LID) [14], sum of edge cluster coefficient (SoECC) [12], and cluster coefficient (ClusterC) [16]. Subsequently, ECDEP's performance is gauged against four shallow machine learning methods combined with RFE, encompassing logistic regression RFE (LR-RFE), SVM-RFE, random forest RFE (RF-RFE), and AdaBoost RFE (AB-RFE). Finally, ECDEP is benchmarked against the deep learning models, DeepEP and MBIEP, which integrate multi-source information. The experimental results affirm ECDEP's supremacy across most datasets, outperforming all comparative methods.

## Materials and methods

### Datasets and preprocessing

The ECDEP model takes inputs from diverse biological data, including PPI network, essential proteins, gene expression profiles, and subcellular localization. The PPI networks are retrieved from a continuously updating database, BioGRID [38], and we also acquire *S. cerevisiae* PPI data from Krogan [39] and DIP [40] databases, which both store accurate verified physical interactions. For all PPI networks, we only adopt physical interactions and subsequently remove self-loops and duplicate records. The basic details of the processed PPI networks can be found in Supplementary Materials: Table S5 To label the samples, experimentally determined essential proteins are required. The essential proteins of *S. cerevisiae*, *M. musculus*, and *C. elegans* are obtained from DEG [41] and OGEE [42] databases, after merging and removing duplicates from these two sources, we remain 1132 *S. cerevisiae* essential proteins, 2914 M*. musculus* essential proteins and 700 *C. elegans* essential proteins. As for *H. sapiens*, referring to the treatment in DeepHE [43], after collecting 20 *H. sapiens* essential protein sets in the DEG database, we consider the protein to be vital if it appears in more than five sets. Detailed data processing is displayed in Supplementary Materials: Table S3.

The Subcellular localization data are attained from the All Channels Integrate column from the COMPARTMENTS [44] database. We consult the approach to handle subcellular localization presented in MBIEP [30] and incorporate slight modifications. Upon ranging the subcellular localization in descending order based on the protein count and selecting the top 1024 ones, we excluded the data in the interval between the top 11 and top 64 due to its minor contribution to the result. Additionally, we individually retrieve GSE3431 [45], GSE41828 [46], GSE3231 [47], and GSE77110 [48] from the GEO database [49], which all possess time course, as the gene expression profiles of *S. cerevisiae*, *H. sapiens*, *M. musculus*, and *C. elegans*. For elaborated processing and information, please refer to Supplementary Materials: Table S4.

### Construction of dynamic PPI network

The Three Sigma Rule (3-sigma) is a common concept in the field of statistics. As displayed in Formula (1 and 2), When applied to calculate the expression data for a given protein p, μ(p) indicates the arithmetic mean of the expression level from time 1 to time n. σ^2^(p) represents the variance of the gene expression level, while EV_i_(p) signifies the expression level at time i.

1 $$\mu \left(p\right)=\frac{{\sum }_{i=1}^{n}{EV}_{1}\left(p\right)}{n}$$

2 $$\sigma^2\left(p\right)=\frac{\sum_{i=1}^n\left({EV}_i\left(p\right)-\mu\left(p\right)\right)^2}{n-1}$$

Recently, the fluctuation of networks gradually received attention in the realm of identifying essential proteins, and gene expression data plays a significant role that providing network dynamics. When studying the relationship between vital proteins and dynamic networks, the 3-sigma rule is the most commonly used tool by researchers [31]. As shown in Formula (3 and 4), F(p) reflects the fluctuation in the expression curve of p, and higher values of σ(p) correspond to smaller values of F(p). The 3-sigma rule aids in assessing whether a protein's expression level at a specific time surpasses the threshold t(p), indicating whether the protein is in an 'active' or 'inactive' state. When a protein is in an active state, it can engage with other active proteins and perform its function [23].

3 $$F\left(p\right)=\frac1{1+\sigma^2\left(p\right)}$$

4 $$t\left(p\right)=\mu\left(p\right)+3\sigma\left(p\right)\left(1-F\left(p\right)\right)$$

**Fig. 1:**
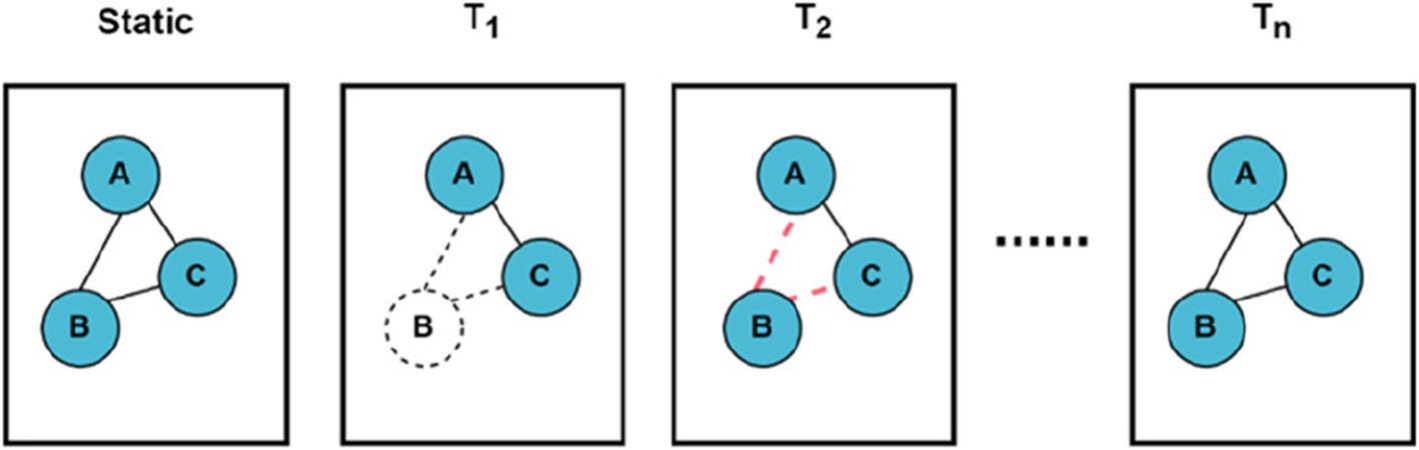
Employ the 3-sigma rule to acquire dynamic networks

Figure 1 illustrates the process of constructing dynamic PPI networks. Initially, in the static PPI network, proteins A, B, and C mutually interact. At every time step, we calculate the threshold t(p) and compare the expression level of each protein. If the expression level of either protein involved in the interaction falls below the threshold, we sever the interaction at this time step.

**Fig. 2:**
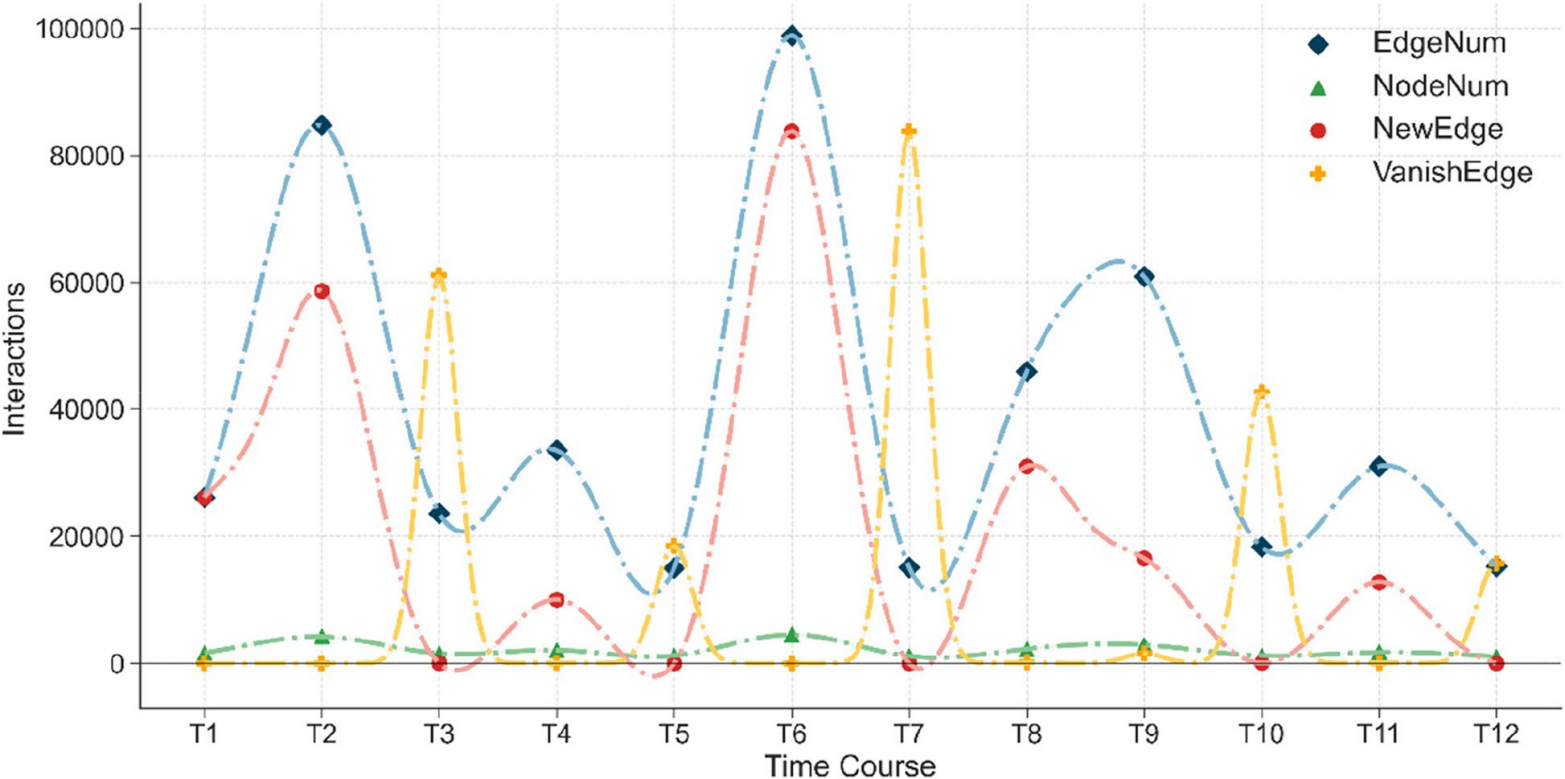
Overview of the temporal evolution of the *S. cerevisiae* PPI network

The expression level of a protein will be decreased after the protein has completed its function which leads to feedback for controlling the expression quantity, while its rate of turnover is constant [50]. We depict the evolving trend of the *S. cerevisiae* PPI network in Fig. 2. Here, EdgeNum and NodeNum denote the current number of edges and nodes in the network, while NewEdge and VanishEdge represent how many edges are generated and disappear at each moment, respectively.

### Evolutional community discovery

The essence of the ECDEP model lies in the utilization of evolutionary community discovery techniques to extract features from dynamic networks and identify cohesive functional modules that emerge during the evolutionary process. While community discovery algorithms are frequently employed in the search for protein complexes, their application in the realm of essential protein identification research remains infrequent.

Due to varying periods utilized in the experimental design of different gene expression data, observation units may range from minutes, hours, days, to weeks, etc., while dynamic community discovery algorithms often demand a higher level of temporal precision [51]. In contrast, evolutionary community discovery primarily concentrates on tracking changes in community structures and is typically applied to networks with more extensive time spans. The primary objective of evolutionary community discovery is to investigate the structure and evolution process of communities, including aspects such as their hierarchical arrangement and modularity. ECDEP model employs the TILES algorithm [32], originally designed for evolutionary community discovery in dynamic social networks, to identify communities in dynamic PPI networks, which effectively detects the emergence and dissolution of communities.

Firstly, we provide a brief definition of the evolutionary community discovery algorithm. Consider an interaction streaming source denoted as S, along with a graph G = (V, E) in which V represents the set of vertices and E signifies the set of edges. Each interaction e ∈ E can be expressed as a triplet (u, v, t), wherein u and v designate the two vertices constituting the edge, and t ∈ N denotes the time step at which this interaction was generated. Evolutionary community discovery aims to consistently identify and update the evolving community structure of graph G in response to the continuous generation of new interactions by S.

Subsequently, we present a basic introduction to the TILES algorithm. TILES operates continuously, analyzing the interaction streaming source S. Upon the generation of a new interaction by S, TILES employs a label propagation mechanism to disseminate this alteration throughout the network, thus refining the community members of the neighborhood. A node can belong to a community with two different levels of involvement: peripheral membership and core membership. Core member participates in at least one triangular relationship with other nodes within the same community. Conversely, nodes that serve as one-hop neighbors to core nodes are referred to as peripheral members. Notably, during the label propagation process, only core members can transmit community membership information to their neighbors. TILES is capable of generating overlapping communities, each delineating different domains and functions.

**Fig. 3:**
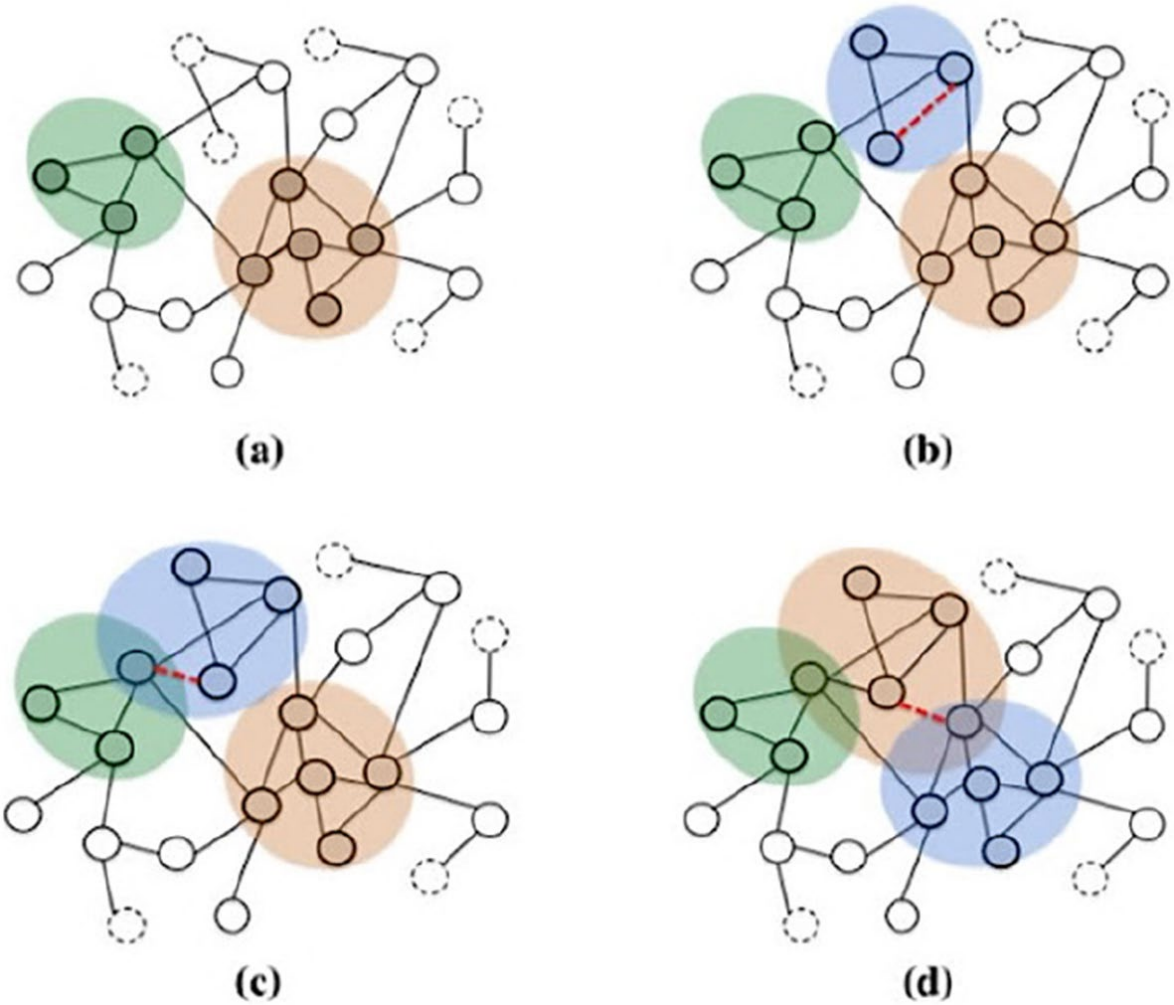
The growth process of TILES communities

**Fig. 4:**
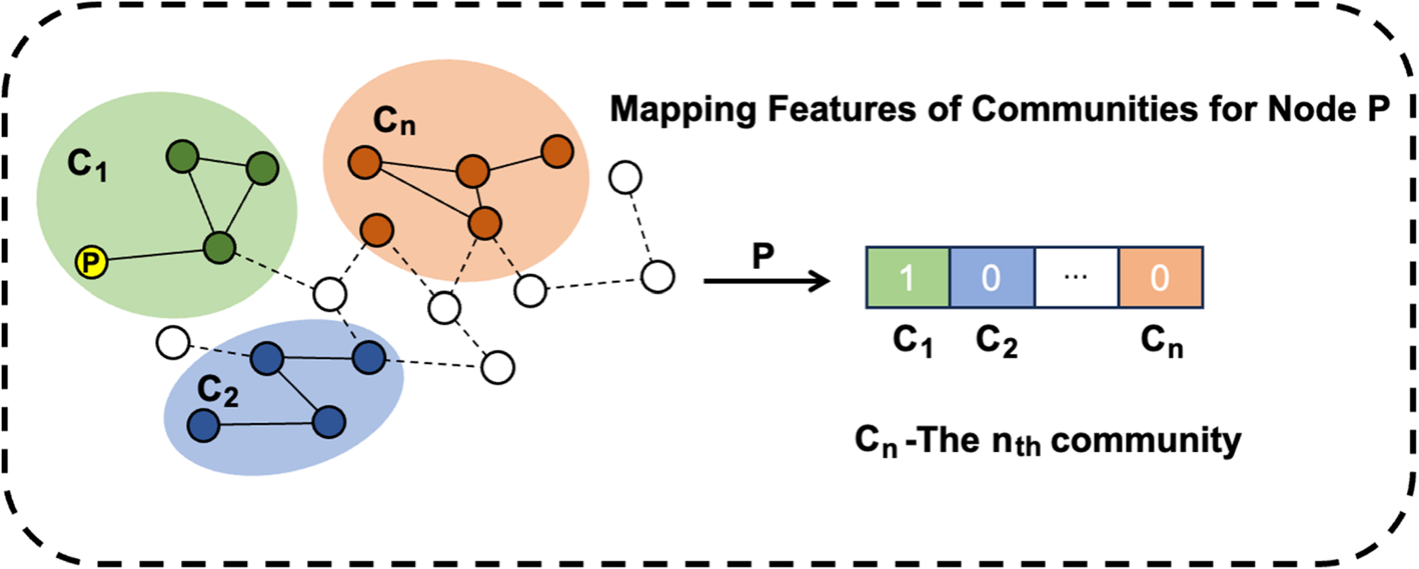
Mapping Feature of Communities for Node P

Figure 3 [32] illustrates the community development process of TILES, where the red dashed lines represent newly added interactions, the colored regions denote distinct core communities and nodes connected by solid external edges without color represent peripheral members. Nodes connected by dashed external edges without color do not participate in any community. The peripheral propagation step is employed to regulate events when new nodes join established communities. If a new node does not form a triangular relationship with any other nodes within the community, it becomes a peripheral member of the community. Additionally, if two existing nodes that do not have any intersecting neighbors interact, the peripheral propagation rule is also applied. However, the core propagation step assumes the existence of at least one common neighbor z between nodes u and v. As depicted in Fig. 3c, for each triplet (u, v, t), if two of the nodes belong to the core members of the same community, the third node also becomes a core member. Otherwise, as shown in Fig. 3a, the algorithm creates a new community based on the triangular relationship. Initially, we integrate communities that have appeared in different observation windows as the initial features using TILES, and subsequently, for each node's features, we represent them using one-hot encoding. We construct an initial matrix with values set to 0 to reflect the presence of nodes in communities. Specifically, each row of the matrix corresponds to a sample, and each column corresponds to a community. If the node in the ${n}_{th}$ row exists in the ${m}_{th}$ community, then the feature matrix element at the nth row and mth column is set to 1; otherwise, it remains 0. As illustrated in Fig. 4, a mapping graph of community features for Node P is depicted.

### Feature selection with SVM-RFE

By employing the TILES algorithm and fine-tuning the observation window, different communities can be generated for each observation interval. These communities may vary in size, exhibit varying degrees of overlap, or even remain entirely disjoint. Consequently, it is important to discern that not all observed communities possess optimal efficacy for the classification task at hand. Therefore, the ECDEP model further utilizes SVM-RFE to enhance the performance of these communities. SVM, coupled with feature selection techniques, has been widely adopted in machine learning-based methods for identifying essential proteins [52].

SVM-RFE is an application of RFE that selects a subset of features by using the weight magnitude as ranking criterion. Given a set of training samples, denoted as **X**_0_ = [**x**_1_, **x**_2_, …, **x**_n_]^T^, along with their corresponding labels, represented as **y** = [y_1_, y_2_, …, y_n_]^T^, firstly, the process commences with the initialization of a subset of surviving features **s** = [1, 2, …, n] and an empty feature ranked list **r** = [].

Firstly, we observed that the initial number of community features was large, and there was significant variability in scale: some communities contained many nodes, while others included only 2 to 3 nodes. Therefore, we decided to employ the SVM-RFE algorithm for feature extraction to retain informative features. Through experimentation, found that selecting 64 features achieved a good balance between model performance and computational efficiency (Supplementary Materials: Table S7). We also attempted to apply SVM-RFE to subcellular localization features. However, the extracted results did not meet the expected performance and were even inferior to using the original features. Consequently, we chose to apply SVM-RFE exclusively to community features. The construction process involves the following steps: ${\mathbf{X}}_{0}$ represents the initial feature set, where ${X}_{n}$ is the community feature vector for each sample. Combining TILES from different observation windows resulted in various strong connected communities, each treated as a feature for the samples, forming a matrix where each row represents a sample, and each column represents a community. As shown in Formula (5), restrict the training samples to ***s***, retaining only the features specified in*** s*** for all samples. Train an SVM classifier with the restricted samples **X** and the corresponding class labels **y**, obtaining the weight vector $\alpha$. Rank the features based on the magnitude of weights in $\alpha$, and remove the features with the smallest weights from the surviving feature subset ***s***. Through iterative iterations, the algorithm gradually eliminates features with minimal impact on classification performance, resulting in an optimized feature subset that enhances model performance and reduces overfitting.

5 $$X=X_0\left(:,\boldsymbol{s}\right)$$

The summations run overall training patterns ${X}_{k}$ that are n-dimensional feature vectors, ${X}_{h}\cdot {X}_{k}$ denotes the scalar product, ${y}_{k}$ encodes the class label as a binary value + 1or − 1. ${\delta }_{hk}$ is the Kronecker symbol (${\delta }_{hk}$= 1 if *h* = *k* and 0 otherwise), its role in the formula is to introduce an additional term between feature vectors. *λ* and *C* are positive constants (soft margin parameters), referred to as regularization and soft margin parameters, respectively. Their presence ensures convergence even in situations where the data is non-linearly separable or poorly conditioned. *C* is a parameter controlling the soft margin, ensuring convergence even in cases of non-linear separability or poor conditions. As illustrated in Formula (6). Given input training samples **X** and their corresponding labels **y**, the weight vector $\alpha$ is obtained through SVM-train.

6 $${\text{SVM}}-\mathrm{train }= \left\{\begin{array}{c}Minimize\;over\;{\alpha }_{k}:\\ J=\left(\frac{1}{2}\right){\sum }_{hk}{y}_{h}{y}_{k}{\alpha }_{h}{\alpha }_{k}({X}_{h}\cdot {X}_{k}+\lambda {\delta }_{hk})-{\sum }_{k}{\alpha }_{k}\\ subject \,to:\\ 0\le {\alpha }_{k}\le C\;and\;{\sum }_{k}{\alpha }_{k}{y}_{k}=0\end{array}\right.$$

As shown in Formula (7), compute the weight vector of dimension length(**s**), the weight vector **w** is a linear combination of training patterns. Most weights ${\boldsymbol{\alpha }}_{{\varvec{k}}}$ are zero. The training patterns with non-zero weights are support vectors. Those with weight satisfying the strict inequality ***0*** < ${\boldsymbol{\alpha }}_{{\varvec{k}}}$< ***C*** are marginal support vectors.

7 $$\mathbf w=\sum\limits_{\mathrm k}{\mathrm\alpha}_{\mathrm k}{\mathrm\gamma}_{\mathrm k}{\mathbf x}_{\mathrm k}$$

For each feature i, compute its ranking criterion ${c}_{i}$, as displayed in Formula (8), and ${w}_{i}$ represents the weight of feature i. Add the feature with the smallest selected ranking criterion to the beginning of the ranking list *r*, as shown in Formula (9). Then, remove the feature with the smallest ranking criterion from the feature set *s*. This process is repeated iteratively, gradually eliminating features with minimal impact on classification performance, resulting in an optimized feature subset, as displayed in Formula (10).

8 $${c}_{i}=(w_i)^2{,} \mathrm{\ for\ all\ i}$$

9 $$\boldsymbol r=\left[\mathbf s\;(\mathrm{argmin}\;(\mathbf c)),\;\mathbf r\right]$$

10 $$\mathbf s=\mathbf s\boldsymbol\;\left(1:\;\mathrm{argamin}\;\left(\mathbf c\right)\;-1,\mathrm{argamin}\;\left(\mathbf c\right)+\;1:\mathrm{linght}\;(\mathbf s)\right)$$

Following this, SVM-RFE computes the ranking criteria and identifies the feature with the smallest ranking criterion to update the feature ranked list, consequently eliminating the feature with the lowest ranking criterion. The iterative training and updating process continues until the set **s** becomes empty, ultimately obtaining a feature list sorted according to the criteria.

### Prediction with ECDEP model

**Fig. 5:**
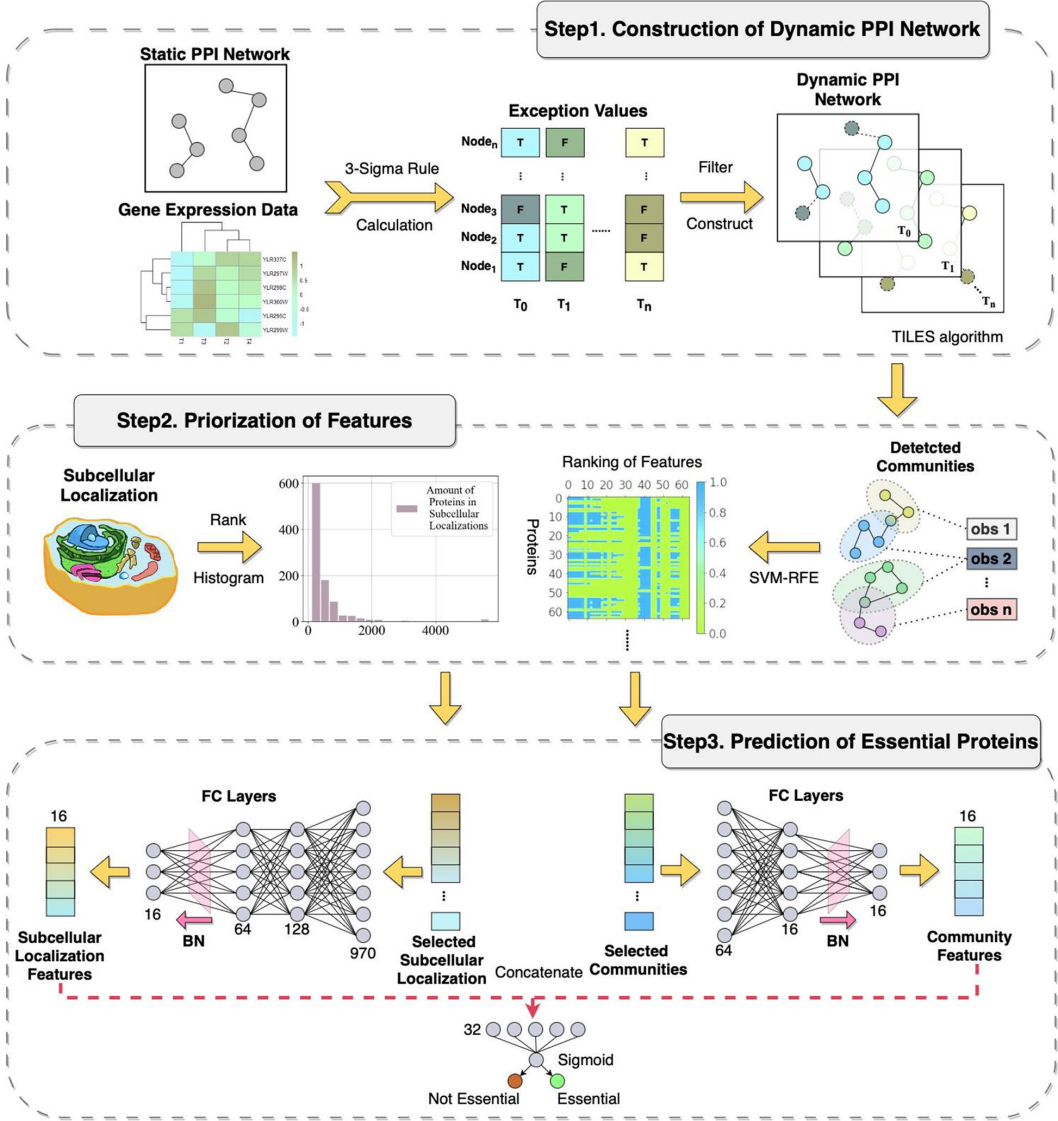
The overall workflow of the EDCEP model. FC Layers: Fully Connected Layers. BN: Batch Normalization Process

Figure 5 provides an overview of the ECDEP deep learning model's architecture, which comprises three primary components: dynamic network construction, feature prioritization, and classification. The model takes three sources of biological information as input, namely subcellular localization, PPI network data, and gene expression data.

In the dynamic network construction phase, ECDEP employs the 3-sigma rule in conjunction with temporal information derived from gene expression data to identify outliers at each time stamp. Subsequently, we merge this information with the static PPI network to capture the network's interaction dynamics at each time point.

Moving on to the feature prioritization step, we utilize an evolutionary community discovery algorithm to extract different overlapping communities from the dynamic network. Following this, we leverage SVM-RFE for the purpose of selecting the most informative communities. These selected communities are then transformed into low-dimensional vectors, serving as node features. When combined with the features extracted from subcellular localization data, these features are input into the final classification module to predict essential proteins.

### Experiment result and analysis

#### Experiment setup

In the process of segmenting the dataset, we allocate 80% of the data for the training set, while reserving the remaining 20% for the test set. Furthermore, to ensure a balanced dataset, we employ a sampling technique in the DeepEP [27]. As depicted in Formula (11), where *M* represents the number of essential proteins, and *N* represents the number of non-essential proteins, with *M* being less than *N*. The probability of selecting a non-essential protein instance is $M/N$. Therefore, for a specific non-essential protein, the probability of not being selected at least once after *k* draws is given by Formula (11). We set the threshold *P* value to 0.001, as a smaller *P* value indicates more effective utilization of all non-essential protein samples, ensuring that no information is lost from the original dataset. Through this sampling technique, we can train with a balanced subset in each training epoch, ensuring that the classifier does not exhibit bias toward any specific class in each training batch. It's important to note that all the machine learning and deep learning methods we compare undergo identical preprocessing steps, thus ensuring the fairness and consistency of the experimental setup.

11 $$p=\left(1-\frac MN\right)^k$$

For details on the experimental environment and package requirements, please access our GitHub repository (https://github.com/LionKingAHAU/ECDEP) or refer to the Supplementary Materials: Table S6. In ECDEP, we utilize binary cross entropy as the loss function and employ ReLU as the activation function. You can find a comprehensive list of other model parameters in Supplementary Materials: Table S7. We conduct tests on various parameter configurations and provide our recommended settings.

#### Comparison with centrality methods

In order to assess the effectiveness of the ECDEP algorithm, we conduct a comparative evaluation with multiple methodologies to gauge the performance and robustness of our model in predicting essential proteins. Centrality methods have long been employed for the identification of essential proteins. Our assessment of centrality methods proceeded as follows: Firstly, compute the centrality value for each protein, sort them in descending order, and then select the top proteins as candidate essential proteins. Subsequently, we evaluate various metrics based on the number of true essential proteins among these candidates.

However, the proportion of essential proteins within a PPI network can vary across different species. In the case of *S. cerevisiae* (BioGRID), *S. cerevisiae* (Krogan), *S. cerevisiae* (DIP), *H. sapiens*, *M. musculus*, and *C. elegans*, the respective proportions of essential proteins are 18.90%, 27.86%, 22.49%, 11.32%, 28.22%, and 8.82%. Consequently, we leveraged these species-specific proportions as the basis for selecting top centrality indices and assessing the performance of the centrality methods.

**Fig. 6:**
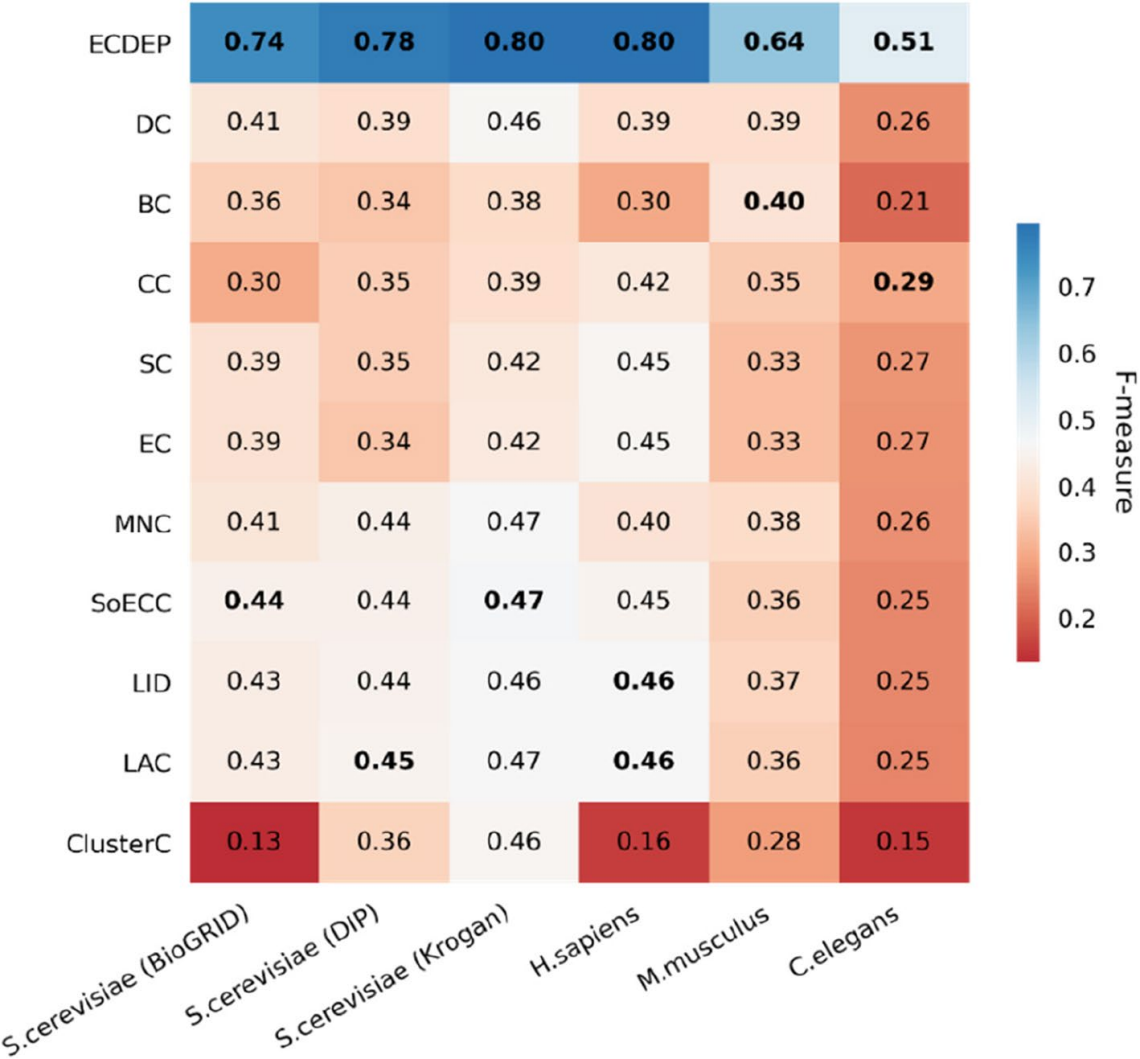
Comparison of ECDEP with centrality methods

Moreover, the proportion of essential proteins from various species leads to the imbalanced learning problem of essential protein identification. Therefore, we primarily select the F-measure as the evaluation metric. The F-measure is the harmonic mean of recall and precision, offering a better reflection of a method's ability to identify positive samples. As shown in Fig. 6, in all comparisons, ECDEP consistently outperforms centrality-based methods and surpasses the performance of SoECC, LAC, LID, BC, and CC, which excel in centrality methods, by margins of 0.30, 0.33, 0.33, 0.34, 0.24, and 0.22, respectively, across the *S. cerevisiae* (BioGRID), *S. cerevisiae* (DIP), *S. cerevisiae* (Krogan), *H. sapiens*, *M. musculus*, and *C. elegans* datasets. Furthermore, the best-performing centrality-based methods vary among different species, revealing the instability of centrality-based approaches, which are influenced by the size and density of networks. Additionally, ECDEP exhibits superior performance in terms of accuracy, precision, and recall compared to all centrality-based methods. Further details can be found in Supplementary Materials: Figure S2. It is anticipated that ECDEP would exhibit superior performance compared to centrality-based methods. ECDEP leverages additional gene expression data and subcellular localization information to mitigate potential noise in the PPI network, and their biological characteristics significantly enhance its effectiveness in the final classification task. In contrast, centrality-based methods rely solely on the topological structure of the PPI network, and some of these methods have demonstrated acceptable results as well. Consequently, when lacking other biological information, centrality-based methods can serve as a viable approach for identifying candidate essential proteins.

### Comparison with machine learning and deep learning methods

To further assess the performance of the ECDEP model, we conduct a comparative analysis involving four shallow machine learning algorithms, one deep learning method DeepEP, utilizing gene expression profiles, and another deep learning method MBIEP, which combines multiple biological information sources.

For the shallow machine learning algorithms, we utilize the same input data as ECDEP and incorporate recursive feature elimination techniques to optimize community features. As for the deep learning methods, the DeepEP model employed GSE3431 as input data, maintaining consistency with our experimental setup for *S. cerevisiae*. However, the gene expression data of other species differ from the *S. cerevisiae* dataset in shape and either short in temporal information or contain extra replicate samples. To ensure uniform input data across all species, we adjust these gene expression datasets according to the parameters specified in the DeepEP model, allowing for consistent formatting. The MBIEP model had more stringent input requirements, necessitating not only gene expression data with temporal characteristics but also requiring different experimental environments and replication samples. Consequently, when evaluating the MBIEP model's performance across the three *S. cerevisiae* datasets, we utilized GSE7645 [53] data. For other biological gene expression datasets, that possess experimental conditions and replication samples, we conduct data preprocessing, including padding and trimming, to meet the input criteria of the MBIEP model.

In comparing our approach with both deep learning and machine learning methods, we primarily relied on F-measure, ROC (Receiver Operating Characteristic), and PR (Precision-Recall) curves to assess method performance. These metrics offer a more objective evaluation of our method's capabilities, especially in the context of imbalanced binary classification problems.

Firstly, we evaluate the performance of ECDEP on the *S. cerevisiae* (BioGRID) dataset (Supplementary Materials: Figure S3). ECDEP achieves an AP value identical to that of MBIEP, while displaying a slight reduction of 0.01 in the AUC value compared to MBIEP. Notably, ECDEP outperforms DeepEP with an AUC value that is 0.12 higher and an AP value that is 0.42 higher. Furthermore, our results indicate that ECDEP's performance surpasses that of all shallow machine learning models included in the comparison. When compared to the DeepEP model, both ECDEP and MBIEP, along with SVM-RFE and LR-RFE, demonstrate superior performance. It is important to highlight that these models extra utilize subcellular localization data as input, resulting in significantly improved predictive capabilities. This underscores the crucial role of subcellular localization information in enhancing the precision of essential protein prediction tasks.

**Fig. 7:**
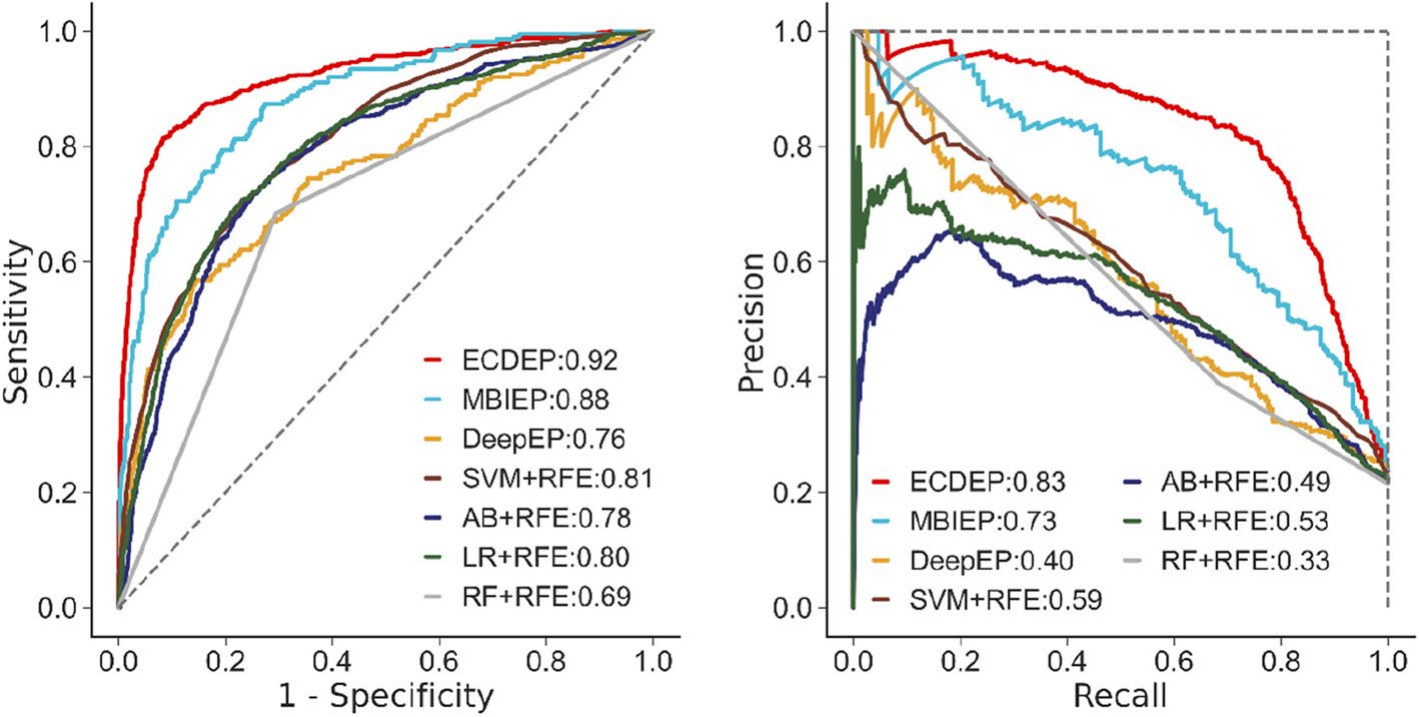
AUC and RP curves of ECDEP compared on the *S. cerevisiae* (DIP) dataset. SVM + RFE: Support Vector Machine with Recursive Feature Elimination; AB + RFE: AdaBoost with Recursive Feature Elimination; LR + RFE: Logistic Regression with Recursive Feature Elimination; RF + RFE: Random Forest with Recursive Feature Elimination

Subsequently, we further assess the performance of the ECDEP model on two additional *S. cerevisiae* databases, as illustrated in Fig. 7. On the *S. cerevisiae* (DIP) dataset, ECDEP exhibits superior performance compared to other methods. Specifically, it achieved AUC and AP values that are 0.04 and 0.10 higher than MBIEP, 0.16 and 0.43 higher than DeepEP, and 0.11 and 0.24 higher than the best-performing SVM-RFE among shallow machine learning methods. Notably, compared to the BioGRID dataset, most algorithms demonstrate a modest improvement in performance. It's worth mentioning that the BioGRID dataset possesses a larger network scale and density, presenting a challenge in effectively leveraging its rich and timely interaction data while mitigating noise.

On the *S. cerevisiae* (Krogan) dataset (Supplementary Materials: Figure S4), ECDEP continues to outperform other models, with AUC and AP values that exceed those of MBIEP by 0.03 and 0.07, DeepEP by 0.15 and 0.43, and the leading SVM-RFE by 0.07 and 0.15, respectively. Additionally, compared to the BioGRID database, the majority of algorithms demonstrated improved performance. The Krogan dataset stands out due to its unique characteristics—all interaction edges are experimentally confirmed, resulting in lower network noise and a higher proportion of essential proteins. This reduces the challenges associated with imbalanced learning in the dataset.

Across all three *S. cerevisiae* datasets, SVM-RFE and LR-RFE emerge as the top-performing shallow machine learning methods, while RF-RFE and AB-RFE present comparatively poorer results. Our experimental approach involves the combination of 970 subcellular localization features and 64 community features into a single one-dimensional vector for input to the machine learning model. According to the results, the input features exhibit a strong linear structure, which contributes to variations in the results.

**Fig. 8:**
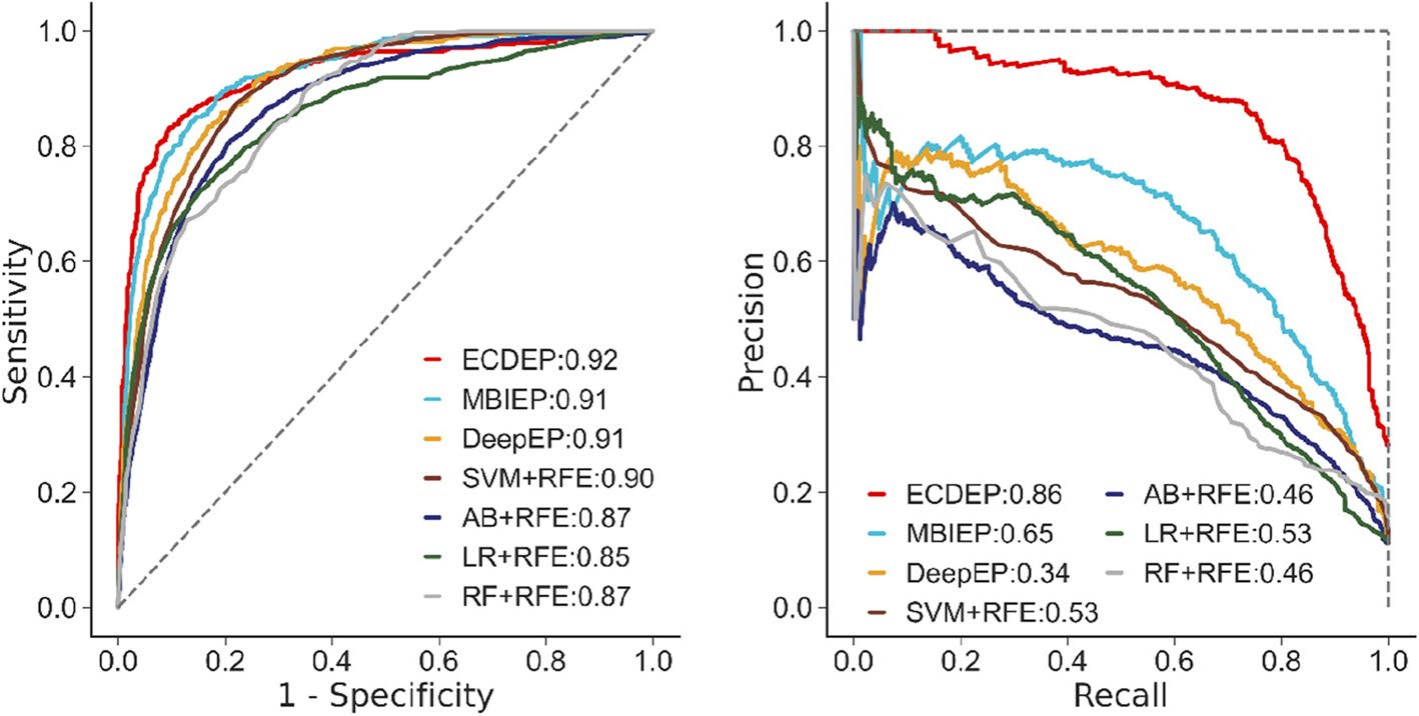
AUC and RP curves of ECDEP compared on the *H. sapiens* dataset. SVM + RFE: Support Vector Machine with Recursive Feature Elimination; AB + RFE: AdaBoost with Recursive Feature Elimination; LR + RFE: Logistic Regression with Recursive Feature Elimination; RF + RFE: Random Forest with Recursive Feature Elimination

In the next step, we investigate ECDEP's performance across different species. Figure 8 depicts a comparative analysis of *H. sapiens* dataset. ECDEP demonstrates notable advantages in this context, with AUC and AP values exceeding those of other models. Specifically, it outperforms the MBIEP model by 0.01 in AUC and 0.21 in AP, surpasses the DeepEP model by 0.01 in AUC and 0.52 in AP, and excels the best-performing SVM-RFE machine learning method by 0.02 in AUC and 0.33 in AP. This enhanced performance is particularly remarkable when contrasted with MBIEP.

It's worth noting that in the MBIEP model, gene expression data are used as feature inputs, but its contribution to classification prediction is relatively low. In contrast, the ECDEP model adopts a novel approach by combining the PPI network and gene expression data, resulting in the creation of community modules exhibiting topological and functional characteristics. This innovative approach significantly enhances the model's performance on this dataset.

Furthermore, it's important to observe that while the differences in AUC values among the algorithms may not be substantial, the differences in AP values are quite significant. This discrepancy arises from the fact that, in the context of the *H. sapiens* species dataset, the proportion of essential proteins is minor, exacerbating the challenge of imbalanced learning. AP values, which emphasize the predictive performance of positive samples, reveal that some comparative algorithms perform poorly on positive categories. Notably, DeepEP exhibits the lowest AP value among the compared algorithms, and it is the sole method lacking subcellular localization input. This observation reveals the critical role of subcellular localization data in predicting essential *H. sapiens* proteins and its substantial contribution to the prediction of positive samples.

**Fig. 9:**
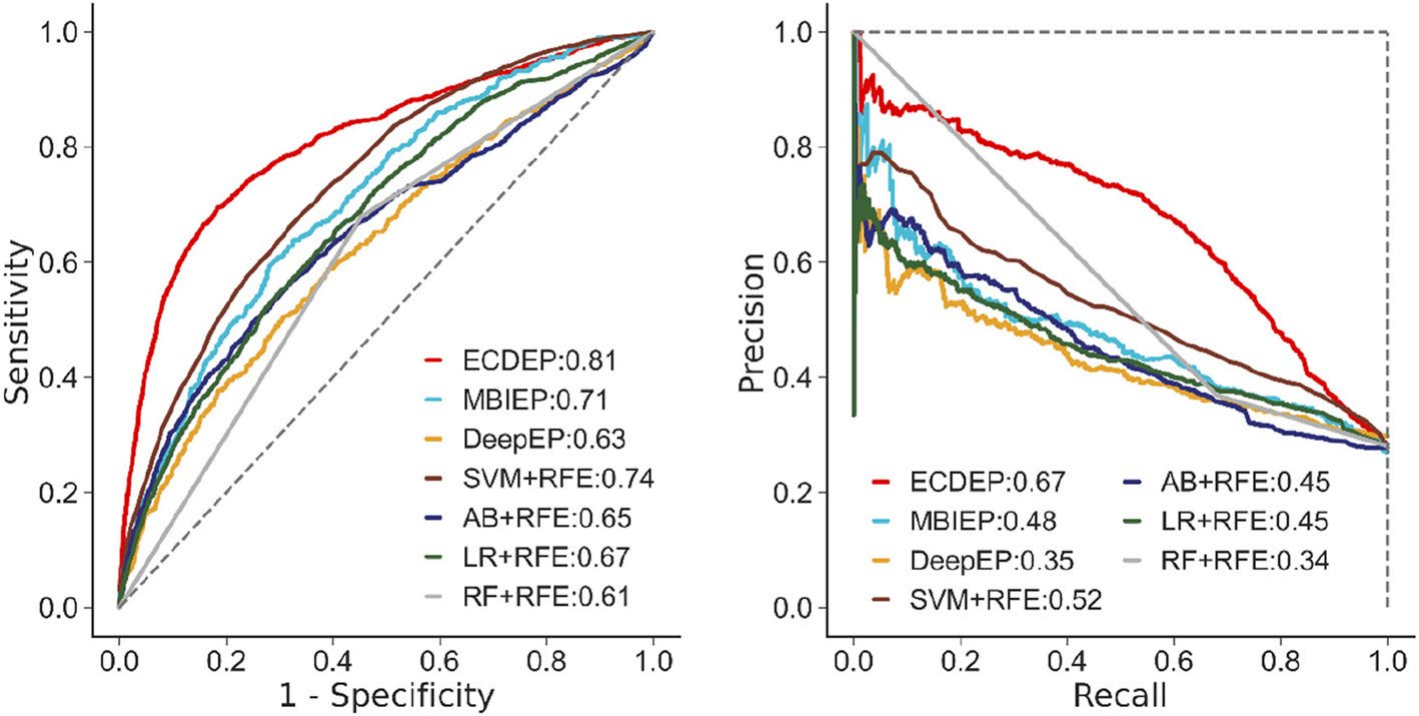
AUC and RP curves of ECDEP compared on the *M. musculus* dataset. SVM + RFE: Support Vector Machine with Recursive Feature Elimination; AB + RFE: AdaBoost with Recursive Feature Elimination; LR + RFE: Logistic Regression with Recursive Feature Elimination; RF + RFE: Random Forest with Recursive Feature Elimination

As follows, we delve deeper into assessing the performance of ECDEP on the *M. musculus* dataset, as illustrated in Fig. 9. ECDEP consistently demonstrates its superiority over all comparative methods, showcasing higher AUC and AP values. Specifically, it outperforms MBIEP by a margin of 0.10 in AUC and 0.19 in AP, and surpasses DeepEP by 0.18 in AUC and 0.22 in AP.

Among the shallow machine learning methods, SVM-RFE emerges as the closest contender, with AUC and AP values trailing ECDEP by a modest 0.07 and 0.15, respectively, while maintaining a slight edge over MBIEP by 0.03 in AUC and 0.04 in AP.

In comparison to the results obtained from *S. cerevisiae* and *H. sapiens* datasets, it is noteworthy that all algorithms experience a minor reduction in AUC values. However, ECDEP and MBIEP stand out due to a notable decrease in AP values, setting them apart from other comparative algorithms, which exhibit minimal fluctuations in AP values.

Our interest lies in understanding the variations in performance on the *M. musculus* dataset, given the high biological similarity between *M. musculus* and *H. sapiens*, including numerous homologous genes. We initially explored different labeling approaches, including applying the DEG database alone, employing the OEGG dataset alone, selecting the intersection of both databases, and choosing data from both homologous genes and essential proteins in both *H. sapiens* and *M. musculus* species. Unfortunately, the experimental results fail to meet our expectations. Therefore, while ECDEP clearly outperforms all comparative methods on the *M. musculus* dataset, there remains an opportunity for further refinement and enhancement.

**Fig. 10:**
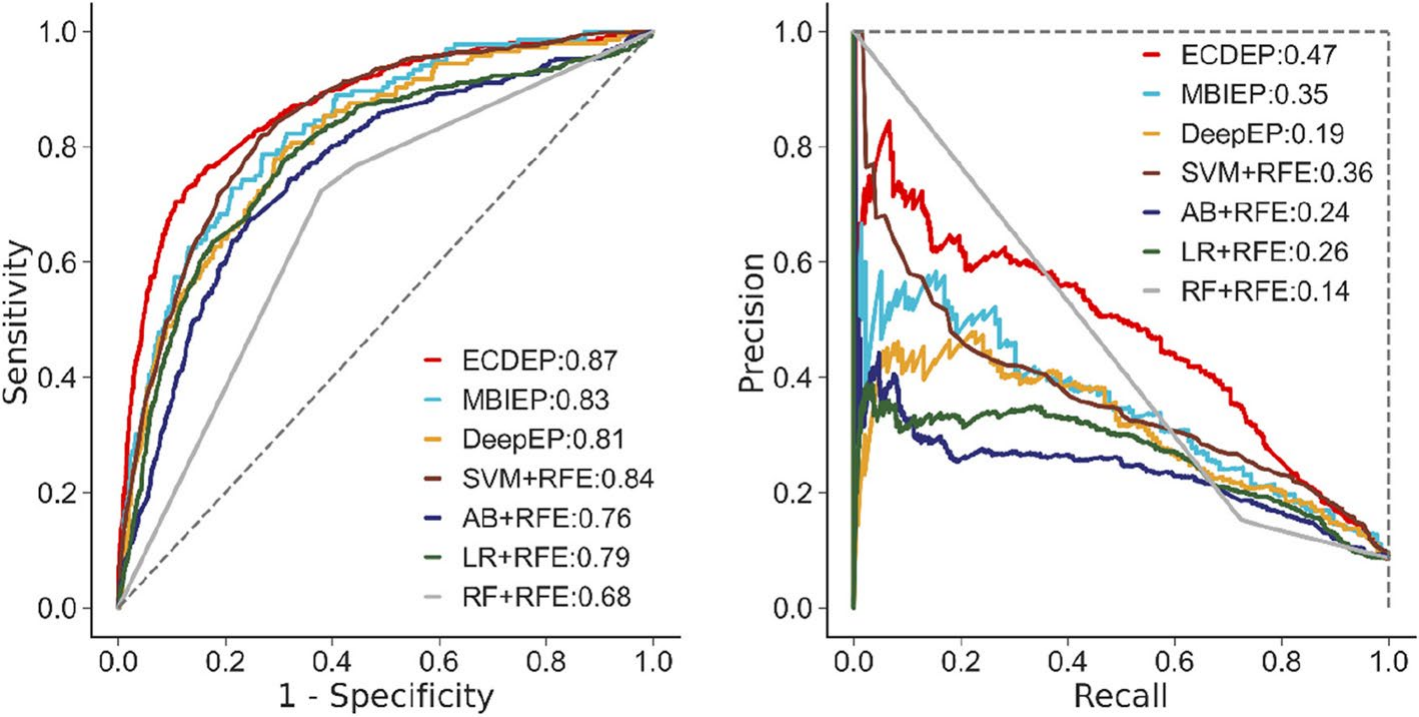
AUC and RP curves of ECDEP compared on the *C. elegans* dataset. SVM + RFE: Support Vector Machine with Recursive Feature Elimination; AB + RFE: AdaBoost with Recursive Feature Elimination; LR + RFE: Logistic Regression with Recursive Feature Elimination; RF + RFE: Random Forest with Recursive Feature Elimination

On the *C. elegans* dataset, as depicted in Fig. 10, ECDEP continues to exhibit its superiority over all comparative methods. It notably surpasses MBIEP, boasting AUC and AP values that are 0.04 and 0.12 higher, respectively. Among the remaining comparative methods, SVM-RFE emerges as the best method, albeit with AUC and AP values trailing ECDEP by 0.03 and 0.11, respectively.

**Fig. 11:**
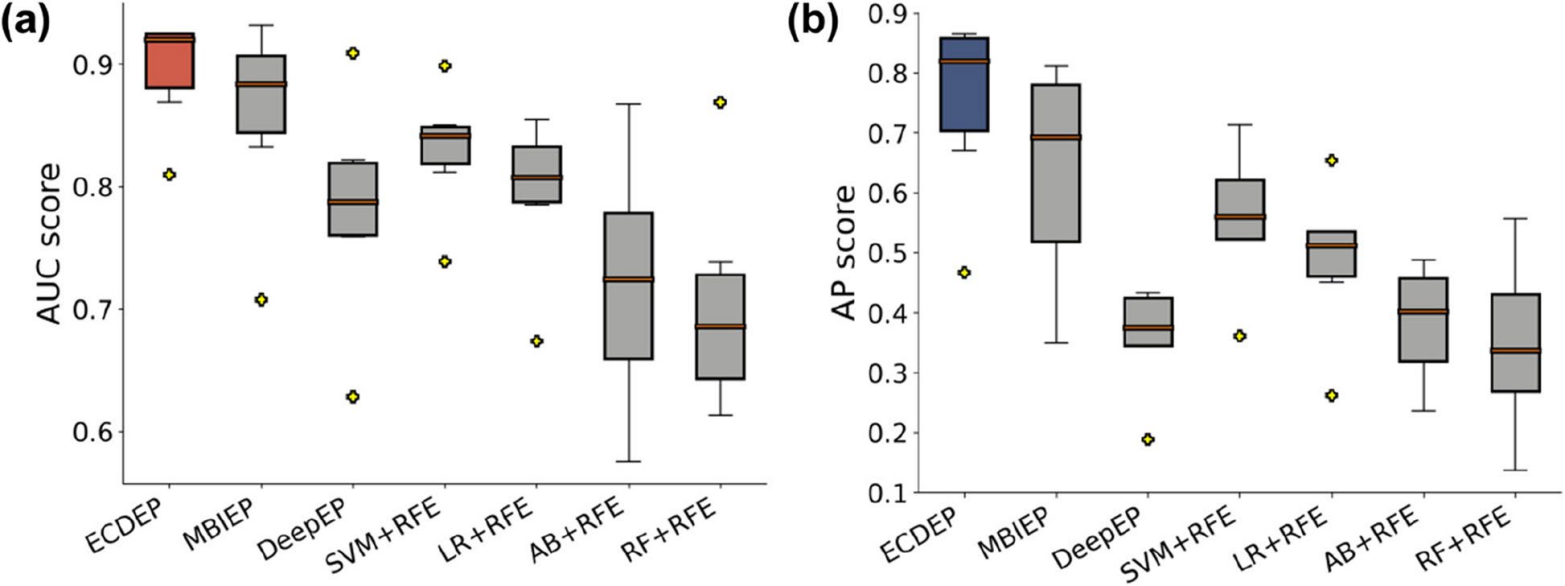
Comparative Analysis of AUC and AP Values. **a** The comparison of AUC values between ECDEP and machine learning methods on different datasets. **b** The comparison of AP values between ECDEP and machine learning methods on different datasets. SVM + RFE: Support Vector Machine with Recursive Feature Elimination; AB + RFE: AdaBoost with Recursive Feature Elimination; LR + RFE: Logistic Regression with Recursive Feature Elimination; RF + RFE: Random Forest with Recursive Feature Elimination

Importantly, all methods experience a significant decline in AP values on the *C. elegans* dataset, which holds the lowest proportion of essential proteins among all datasets, exacerbating its imbalanced learning challenge. Upon comparison, we find that despite its lower proportion of essential proteins, the *C. elegans* dataset yields a relatively higher AUC values for the algorithms. This phenomenon can be attributed to the dataset's elevated ratio of negative samples, enabling the models to correctly identify more negative instances. However, the models struggle to accurately detect positive samples, resulting in high AUC but low AP scores. From Fig. 11a and b, it is evident that ECDEP outperforms all other machine learning models.

Similarly, while ECDEP maintains its superiority over all comparison algorithms on the *C. elegans* dataset, its performance has markedly declined, which can be ascribed to the dataset's limited number of positive samples and the inherent differences between species.

When we evaluate the performance of the ECDEP model across the four species, we observe that it excels on *S. cerevisiae* and *H. sapiens* datasets but experiences performance degradation on *M. musculus* and *C. elegans* datasets. Several factors contribute to this phenomenon, In addition to the impact of imbalanced datasets, essential protein labels, and differences between different species, there is also the selection of gene expression data. In this study, gene expression data with temporal settings, such as the GSE3231 gene expression data for embryonic stem cell differentiation and the *C. elegans* time course study on binary restriction and aging, are included to provide temporal information. While these datasets were not directly employed as sample features and the utilization of evolutionary community discovery methods effectively leveraged PPI networks and gene expression data, nonetheless, these experiments had a one-sided focus to some extent, resulting in relatively limited functional communities within the dynamic network, contributing to the decline in model performance.

**Fig. 12:**
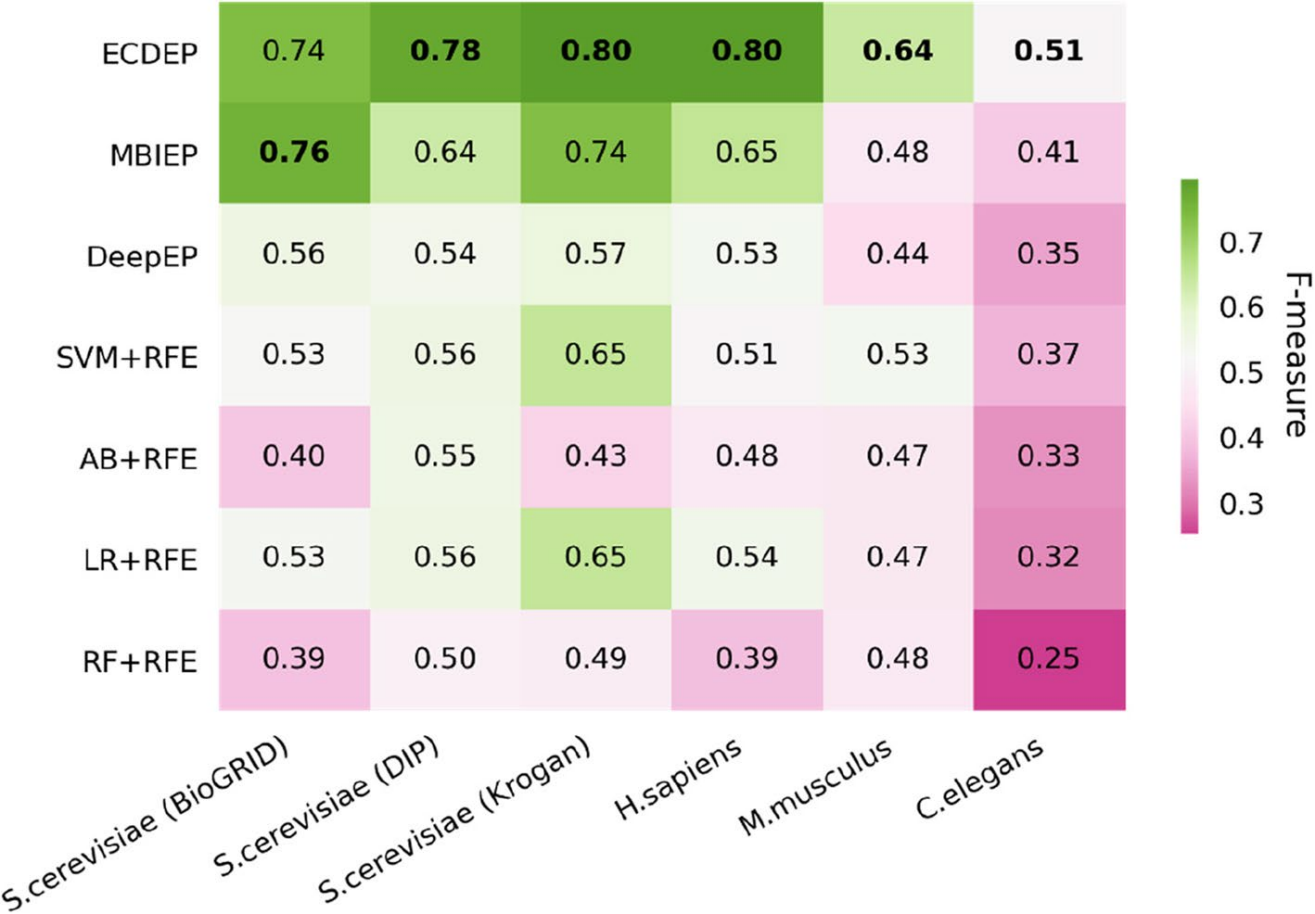
Comparison of ECDEP with machine learning methods. SVM + RFE: Support Vector Machine with Recursive Feature Elimination; AB + RFE: AdaBoost with Recursive Feature Elimination; LR + RFE: Logistic Regression with Recursive Feature Elimination; RF + RFE: Random Forest with Recursive Feature Elimination

Figure 12 presents F-measure evaluation metrics for ECDEP and comparative algorithms across various datasets. On the *S. cerevisiae* (BioGRID) dataset, ECDEP's F1 value falls 0.02 short of MBIEP's performance. However, on the *S. cerevisiae* (DIP), *S. cerevisiae* (Krogan), *H. sapiens*, *M. musculus*, and *C. elegans* datasets, ECDEP outshines all comparison methods, boasting F1 values that surpass MBIEP by 0.14, 0.06, 0.15, 0.16, and 0.10, respectively. This demonstrates ECDEP's remarkable ability to identify positive samples across diverse datasets.

We conduct a more comprehensive comparison of ECDEP's performance on other metrics (Supplementary Materials: Figure S8). While ECDEP's accuracy on the *H. sapiens* dataset lags behind MBIEP by 0.03, it consistently achieves the highest accuracy across other datasets. Moreover, ECDEP consistently exhibits the highest precision on all datasets, except for recall values where it performs merely better on *S. cerevisiae* (DIP) and *S. cerevisiae* (Krogan) datasets. Conversely, LR-RFE, RF-REF, and SVM-RFE demonstrate the highest recall values across other datasets. This observation suggests that ECDEP tends to be more conservative in identifying positive samples, attributing to its higher credibility in positive sample identification, albeit potentially leading to some misclassification of positive samples as negative.

In contrast, shallow machine learning methods, while achieving high recall values, often compromise on precision, leading to misclassification of negative class samples as positive. Taking into account a comprehensive F-measure evaluation, the ECDEP model emerges as the most suitable choice for this task, balancing precision and recall effectively.

### Comparison with ensemble learning and graph convolutional network methods

**Fig. 13:**
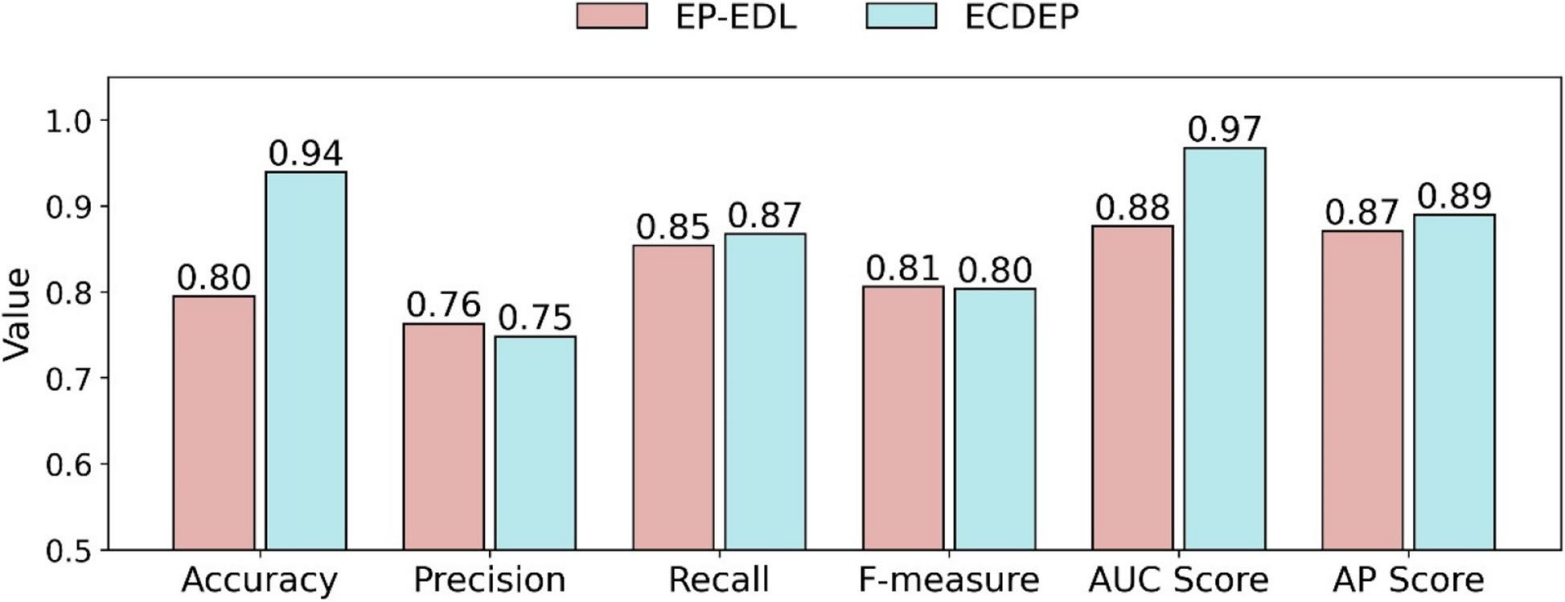
Compare ECDEP with EP-EDL method with various metrics on the Intersection Proteins dataset

There's currently a rise in methods that precisely predict essential proteins or genes solely based on sequence information, which is more available. These methods extract patterns from sequences to capture the characteristics of essential proteins. To ensure the superiority of our approach, in this section, we compared it to a method, EP-EDL [54], which relies on protein sequences and ensemble learning for accurate prediction of essential proteins. To ensure a fair comparison basis, we aimed to conduct the comparison on the same protein dataset. As our dataset is significantly larger than EP-EDL's, we performed a match and ID mapping between the datasets using Uniprot IDs. However, within the EP-EDL dataset, there are 1045 proteins that are not included in our dataset. Therefore, as illustrated in Supplementary Fig. 13, we opted to select the intersection of essential proteins and non-essential proteins present in both EP-EDL and ECDEP datasets. Due to the utilization of different datasets, we conducted retraining and testing. For EP-EDL, following the hold-out strategy outlined in its paper, we independently partitioned 20% of the essential proteins and an equal number of non-essential proteins, forming a balanced dataset of positive and negative samples. All other settings remained unchanged. For ECDEP, we re-filtered the PPI network, constructed the dynamic network, and extracted features based on the new dataset, while keeping the remaining parameters and configurations constant. To ensure a comprehensive comparison, as depicted in Fig. 13, we present a comparison of different evaluation metrics. Our proposed method exhibits higher scores by 0.14 in Accuracy and 0.09 in AUC scores compared to EP-EDL. However, the differences in other metrics are marginal, with only a lead or lag of one to two points. Nonetheless, considering an overall perspective, our proposed method still outperforms the EP-EDL method.

Given the rapid advancements in graph convolutional neural networks and the specific characteristics of our targeted task, we endeavored to address the node classification challenge within PPI networks using classical graph convolutional networks (GCNs) [55] on the S. cerevisiae (BioGRID) dataset. However, the outcomes did not align with our initial expectations. We explored the incorporation of community features, subcellular localization features, and a fusion of both as node attributes. Two-layer and three-layer GCNs were separately applied to these distinct feature sets. Unfortunately, the achieved results fell short of our anticipated performance benchmarks. Based on our experimental findings, we hypothesize that integrating community features within the GCN framework may not be conducive to enhanced performance. Additionally, the high dimensionality of subcellular localization features as node attributes might have adversely impacted model efficacy. Consequently, attempts to reduce the dimensionality of subcellular localization features using SVM-RFE, condensing the initial 970-dimensional space to 256 dimensions, yielded unsatisfactory results. As shown in Supplementary Materials: Figures S15, we utilized AUC and AP scores as evaluation metrics. The performance of methods based on graph convolutional neural networks was significantly lower than that of the ECDEP method, which caught our attention. We speculate that this outcome may be due to imbalanced sample categories. We intend to delve deeper into this issue in our future research endeavors.

### Ablation study in ECDEP

#### Contributions from different features

**Fig. 14:**
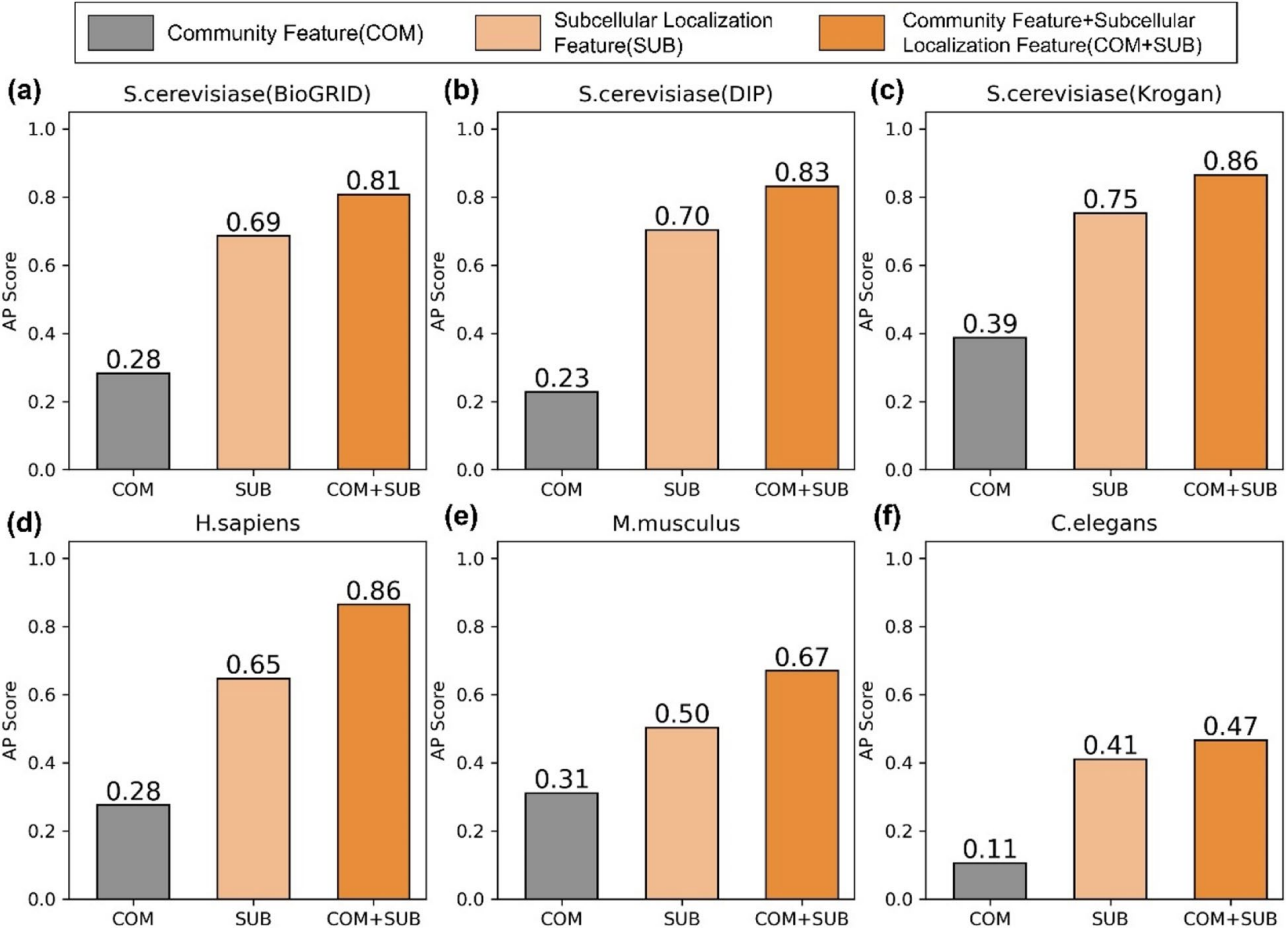
Ablation study of features in ECDEP across six datasets evaluated with AP score. **a f** exhibit different species and datasets. COM: community feature; SUB: subcellular localization feature; COM + SUB: community feature combined with subcellular localization feature

The ECDEP model takes community extracted from dynamic networks and subcellular localization data as its inputs. To ensure fairness and comparability, both sets of features undergo processing through fully connected layers, resulting in one-dimensional vectors with a length of 16. To gain a deeper understanding of the relative contributions of these two input sources to the final classification across different species, for the subcellular localization and selected community features, we individually processed these two types of features using modules within ECDEP. We then separately fed them into the classification layer for predicting essential proteins. As depicted in Fig. 14, we primarily used the area under the precision-recall curve as the primary evaluation metric, comparing six different datasets. In the figure, the grey bars represent training and prediction solely using selected community features, the light orange bars represent training solely with subcellular localization features, and the dark orange bars represent training using a combination of subcellular localization and community features, which represents the results of the ECDEP model. It is evident that the subcellular localization data significantly contributes more to the prediction tasks across different datasets. While the contribution of community features is comparatively lower, its inclusion elevates the AP score by varying degrees, ranging from 0.06 to 0.21. Therefore, we believe that in this model, both features are indispensable. In Supplementary Materials: Figures S9, we provide a comparison of the two features based on the area under the AUC curve. The inclusion of community features similarly enhances the AUC score by 0.12 on the M. musculus dataset, further demonstrating the indispensability of community features. These findings align with our previous results. Although gene expression data are not directly employed as sample features, the dynamic network it participates in constructing and the community modules it captures may exhibit some bias or limitations. Consequently, while the ECDEP model has made strides in enhancing the role of gene expression data in prediction, it remains clear that this feature's contribution alone is not sufficient to achieve optimal results.

#### Benefits of using various techniques

To enhance the precision of predicting essential proteins, this study employs a series of key techniques and strategies. Initially, in light of the prevalent class imbalance in biological datasets, we implement down-sampling techniques to ensure that the model avoids favoring the majority class in its predictions. This effectively mitigates the risk of the model overly optimistic in identifying nonessential proteins, ultimately contributing to the model’s improved generalization capability. Moreover, we leverage RFE techniques to proficiently address the complexities inherent in biological network communities while ensuring consistent input data shapes. The development of community modules within dynamic networks demands extensive feature engineering to aptly model intricate community structures. RFE serves a dual purpose by reducing dimensionality and preserving input data uniformity, thereby enhancing model scalability and generalizability. These integrated strategies empower our model to realize significant performance enhancements across diverse biological datasets.

**Fig. 15:**
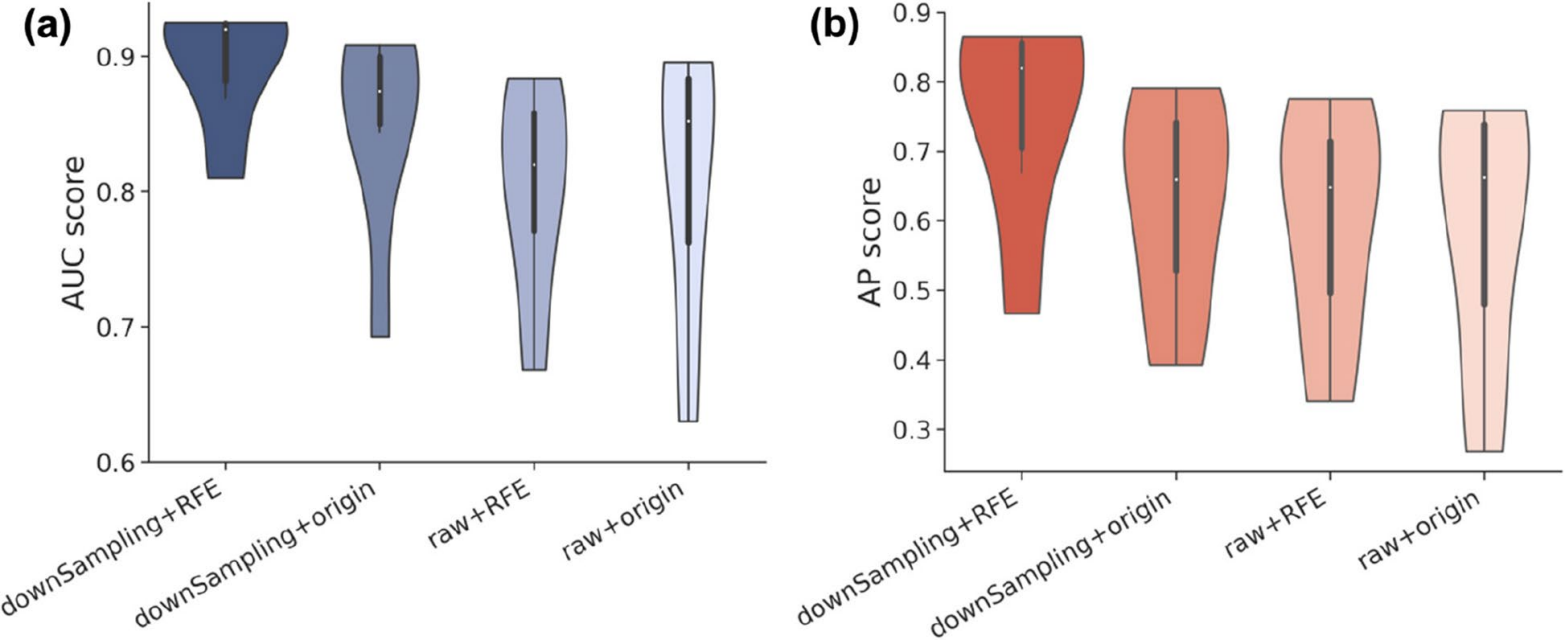
Comparative analysis of AUC and AP values for downsampling and RFE experimental results. **a** AUC values comparison violin plot for downsampling and RFE experimental results. **b** AP values comparison violin plot for downsampling and RFE experimental results

The Fig. 15 illustrates the substantial positive influence on the model’s performance when employing downsampling, as reflected in AUC and AP values. This is attributable to downsampling’s ability to rectify class imbalance challenges within biological datasets, effectively preventing the model from developing a strong bias toward the majority class. Consequently, it reduces the likelihood of erroneous identifications of non-essential proteins, thus amplifying the model’s generalization proficiency. Downsampling markedly bolsters the model’s capacity to address imbalanced data, resulting in an overall boost in its generalization prowess. Furthermore, the strategic utilization of RFE proves instrumental in navigating the intricate landscape of biological network communities, contributing to an augmented model performance. As RFE diminishes feature count, it simultaneously preserves uniformity in input data shapes, thereby enhancing model scalability and generalization capabilities. RFE plays a pivotal role in managing the intricacies of community structures and feature engineering, exerting a constructive influence on the model’s performance. Conversely, the deployment of raw data appears to have a somewhat subdued positive impact on the model’s performance when contrasted with the utilization of pre-processed data. This phenomenon may be attributed to the fact that the pre-processing and feature selection applied to biological data can effectively eliminate noise and redundancy, elevating data quality and aiding the model in capturing indispensable features. A notable aspect of our data analysis is that under the raw + RFE combination, AUC values appear lower in comparison to the raw + origin combination. This could be attributed to RFE’s capability to reduce data dimensionality, albeit occasionally resulting in the elimination of pivotal features, thereby potentially adversely affecting the model’s performance. The raw + RFE combination might exclude some valuable features necessary for the classification task, consequently leading to a reduction in AUC values.

#### Detachment of every state in dynamic network

We further examined whether each snapshot of the dynamic network contributes to the final outcome. Therefore, as illustrated in Supplementary Materials: Figures S10, we took the example of S. cerevisiae (Krogan) and sequentially detached its 12 states. The ECDEP process was then executed using the remaining 11 snapshots. Keeping the model's parameters and other settings constant, a total of 12 models were trained to observe the impact after extracting each snapshot. As shown in Supplementary Materials: Figures S11, we used the F1 score, AUC score, and AP score to measure the results. The red dashed line in the graph represents the results from ECDEP, depicting the overall evolutionary process in the entire graph. The x-axis ranges from T1 to T12, representing the training and prediction results after detaching the snapshots at those time points. The extraction of snapshots resulted in lower F1 and AP scores compared to ECDEP’s results. The F1 score was at its lowest after extracting snapshot T11, experiencing a decrease of 12.16% compared to ECDEP. The AUC and AP scores were lowest after extracting snapshot T12, decreasing by 6.43% and 13.23%, respectively, compared to ECDEP. However, the differences in results were relatively small when extracting snapshots at T2 and T7. Interestingly, the impact of network extraction at different time points did not show a clear correlation with the timeline concerning the final outcome. Furthermore, contrary to our expectations, the extraction of the first snapshot did not result in the worst outcome. In the process of constructing the interaction streaming source of ECDEP, the behavior at the first time point involves generating all edges of the static network. Therefore, the first snapshot represents the static PPI network, upon which the subsequent networks are constructed. As a result, the first snapshot contains the most comprehensive interaction information. However, according to results, its extraction did not yield the worst outcome. In conclusion, each state appears to be indispensable for the final predictive outcome.

#### Dynamic network versus static network

We utilized the first snapshot which contains the most comprehensive edge information to represent other snapshots. The first snapshot is the static PPI network obtained from public databases. In static PPI networks, node2vec [28] is the most commonly used method for extracting features, such as DeepEP [27], Zeng's method [56], and DeepHE [43]. Therefore, in this comparison section, we employed node2vec to extract both the topological and semantic features of the network. The output embedding dimension was set to 64, allowing each protein node to have a 64-dimensional feature. This dimension matches the community feature dimension extracted from the dynamic network in ECDEP. we kept the other parts of the ECDEP model unchanged, replaced the input features of the community module with the embedding features from node2vec, and conducted training and predictions. As shown in Supplementary Materials: Figures S12, we used the F1 score, AUC score, and AP score to evaluate the two methods. In the figure, we used the prefix 'static_' to describe features extracted from the static network and 'dynamic_' to describe the results from ECDEP. The results indicate that the embedding features extracted based on node2vec experienced significant decreases in F1 and AP scores across different datasets. Particularly, on the M. musculus dataset, the dynamic network method outperformed the static network method by 18.95% in the F1 score, 17.38% in the AUC score, and 23.81% in the AP score.

#### Utilizing temporal methods for dynamic network

The ECDEP method relies on the TILES algorithm to extract communities that have emerged during the evolutionary process of the graph. However, the sequential temporal characteristics of the graph also warrant exploration, specifically delving into the trends in network evolution over time. Therefore, in this section, we explicitly leverage the temporal properties of dynamic graphs. Taking the S. cerevisiae (Krogan) dataset as an example, we extract both topological and semantic information of nodes in twelve snapshot networks using node2vec, resulting in (12, 64)-dimensional features for each node. Subsequently, we employ GRU [57], BiGRU [58], LSTM [59], and BiLSTM [60] for feature extraction. Afterward, the outputs are flattened and fed into a fully connected layer, yielding a 16-dimensional vector. Additionally, we maintain consistency in subcellular localization handling, keeping other experimental settings consistent with ECDEP. We measure using AUC and AP scores, as depicted in Supplementary Materials: Figures S14. The performance differences among these RNN-based methods are marginal, none of them outperforming the ECDEP method. They lag behind the ECDEP method by only one to six points in AUC scores but by seven to nine points in AP scores. We speculate that our approach to handling dynamic networks may not be suitable, leading to discrepancies between the results and expectations. In the future, we aim to explore solutions using dynamic graph convolutional networks or evolutionary graph convolutional networks to address this issue.

### Exploration of the incompetent of machine learning methods

The primary reasons for the relatively poorer performance of machine learning methods compared to deep learning methods can be attributed to two main factors: feature characteristics and the inherent attributes of machine learning.

### Concentrate feature with dense layers

The imbalance in feature lengths can introduce bias into the results. In this scenario, subcellular localization features have a length of 970, while community features extracted from gene expression data are limited to a length of 64. This disparity in feature lengths can impact the performance of machine learning models, as these models may exhibit a preference for handling longer features while overlooking shorter ones. Machine learning models are generally sensitive to feature lengths, and in this specific case, the feature lengths may not be conducive to optimal machine learning model performance. The imbalance in feature lengths and the presence of longer features can pose challenges for machine learning models during data analysis. To address this issue, the researchers made a deliberate choice to employ machine learning as the final classifier within the ECDEP model, thereby allowing deep learning methods to harness their strengths more effectively. Considering the feature length imbalance and the inherent characteristics of machine learning models, the decision to utilize machine learning as the concluding classifier within the ECDEP model was made. This strategic move serves to ensure that the model can proficiently manage these unique data attributes, leading to enhanced performance and robustness for the more accurate prediction of essential proteins.

**Fig. 16:**
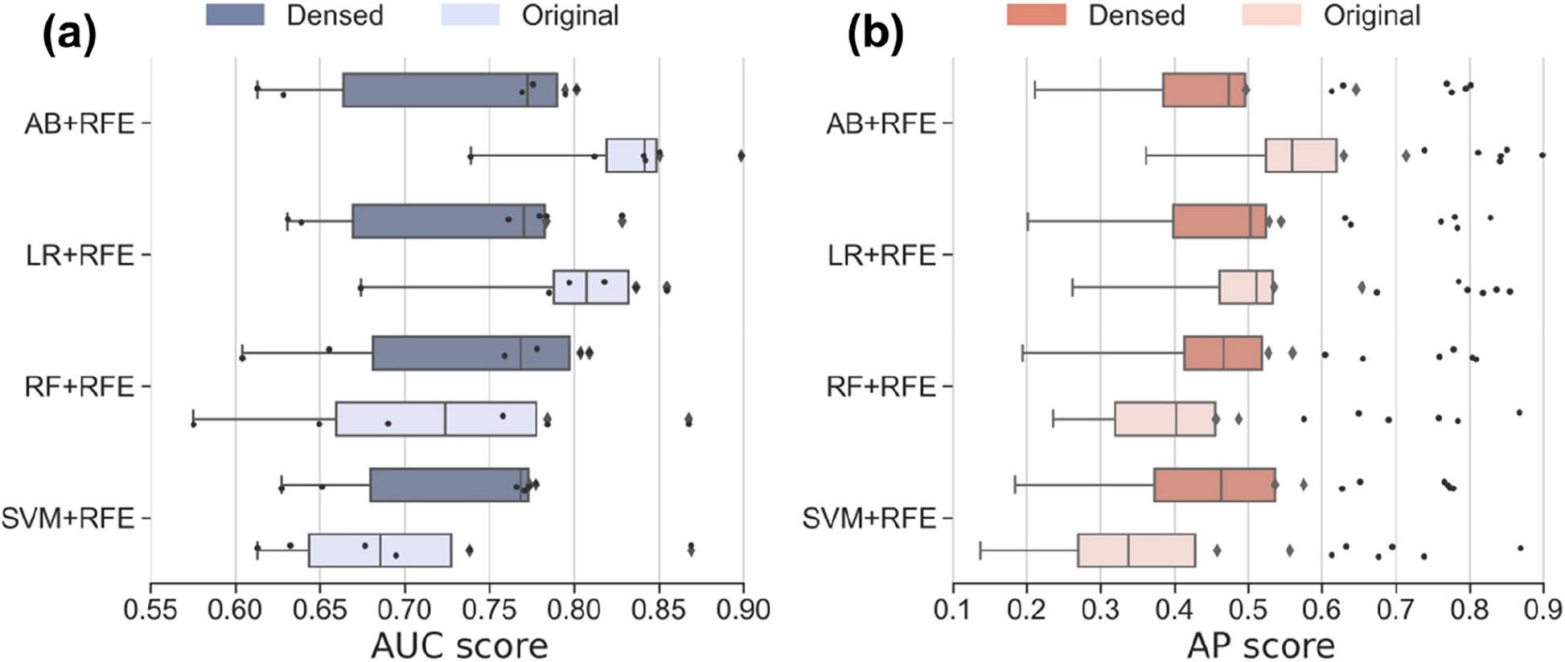
Performance evaluation of machine learning models after densed. **a** AUC value of machine learning model after densed. **b** AP value of machine learning model after densed. SVM + RFE: Support Vector Machine with Recursive Feature Elimination; AB + RFE: AdaBoost with Recursive Feature Elimination; LR + RFE: Logistic Regression with Recursive Feature Elimination; RF + RFE: Random Forest with Recursive Feature Elimination

As illustrated in the Fig. 16, a clear trend emerges when the models are trained using the fully connected layer. The results reveal a decrease in performance for SVM and LR, while AB and RF demonstrate improvements. It’s important to note that SVM and LR are linear models designed for handling linearly separable or nearly linearly separable data. In contrast, AB and RF are ensemble learning models known for their enhanced capacity to handle non-linear relationships. The fully connected layer, however, specializes in learning non-linear feature representations, which diverge from the fundamental principles of linear models. Consequently, the introduction of additional non-linearity through the dense layer may lead to a decline in feature performance for the linear models.

### Use PCA for Feature Dimensionality Reduction

**Fig. 17:**
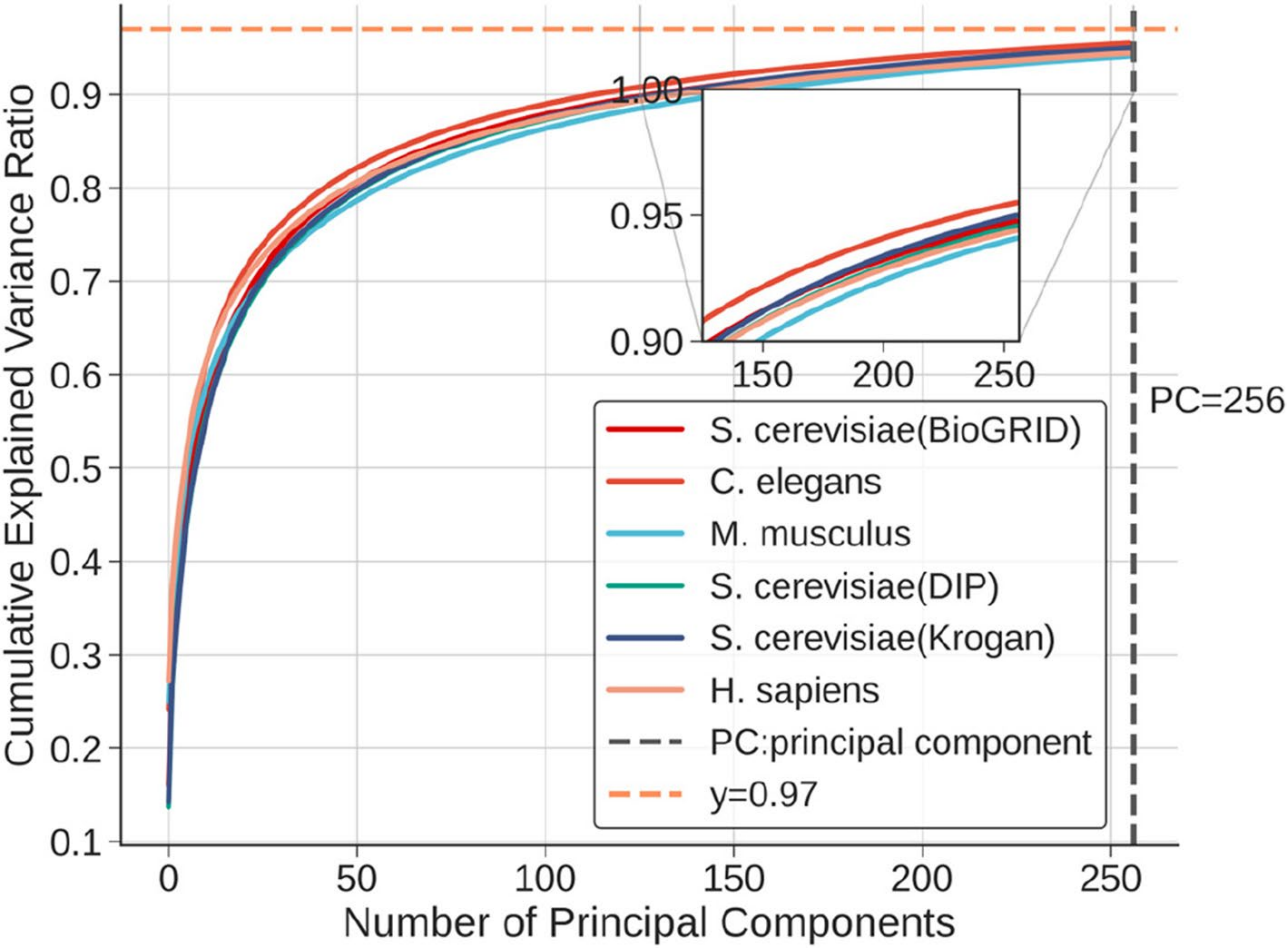
PCA process on subcellular localization features across diverse datasets

We applied Principal Component Analysis (PCA) as a dimensionality reduction technique tailored for subcellular localization analysis. The process of PCA entails the extraction of principal components from the original features to represent the data while minimizing information loss. The results of our experiments exhibit a distinct pattern, as seen in Fig. 17, which aligns with the observations described above.

The sharp increase in cumulative explained variance, as depicted in the initial part of the curve, emphasizes the potency of a small number of principal components. These components encapsulate a substantial portion of the variance within the data, signifying their role in capturing major patterns and structures. We can infer that these initial components are responsible for conveying the most influential information related to subcellular localization. Conversely, the slow convergence of the cumulative explained variance curve, as more principal components are added, suggests a critical aspect of our approach. It indicates that we are progressively incorporating components that capture less significant variance in the data. However, the retained components continue to encapsulate the essential information that characterizes subcellular localization patterns, ensuring that we do not overlook essential protein insights within the data. Despite these advantages, it is important to heed certain caveats when employing dimensionality reduction techniques like PCA in biological data analysis. One such consideration is the potential for information loss. By eliminating some components, we run the risk of removing subtle but important patterns or information embedded in the data, as indicated in the’Information Loss’ section. This implies that while PCA is effective in reducing dimensionality, it should be applied judiciously to avoid discarding valuable data insights.

Principal components are linear combinations of the original features, making their direct interpretation challenging. Researchers need to consider this aspect while employing PCA and should be aware that interpreting the meaning of each component might not always be straightforward. Our application of PCA to subcellular localization data mirrors the findings described in the literature. The tradeoff between dimensionality reduction, information loss, and interpretability underscores the need for a balanced approach when implementing techniques like PCA in the analysis of complex biological datasets.

**Fig. 18:**
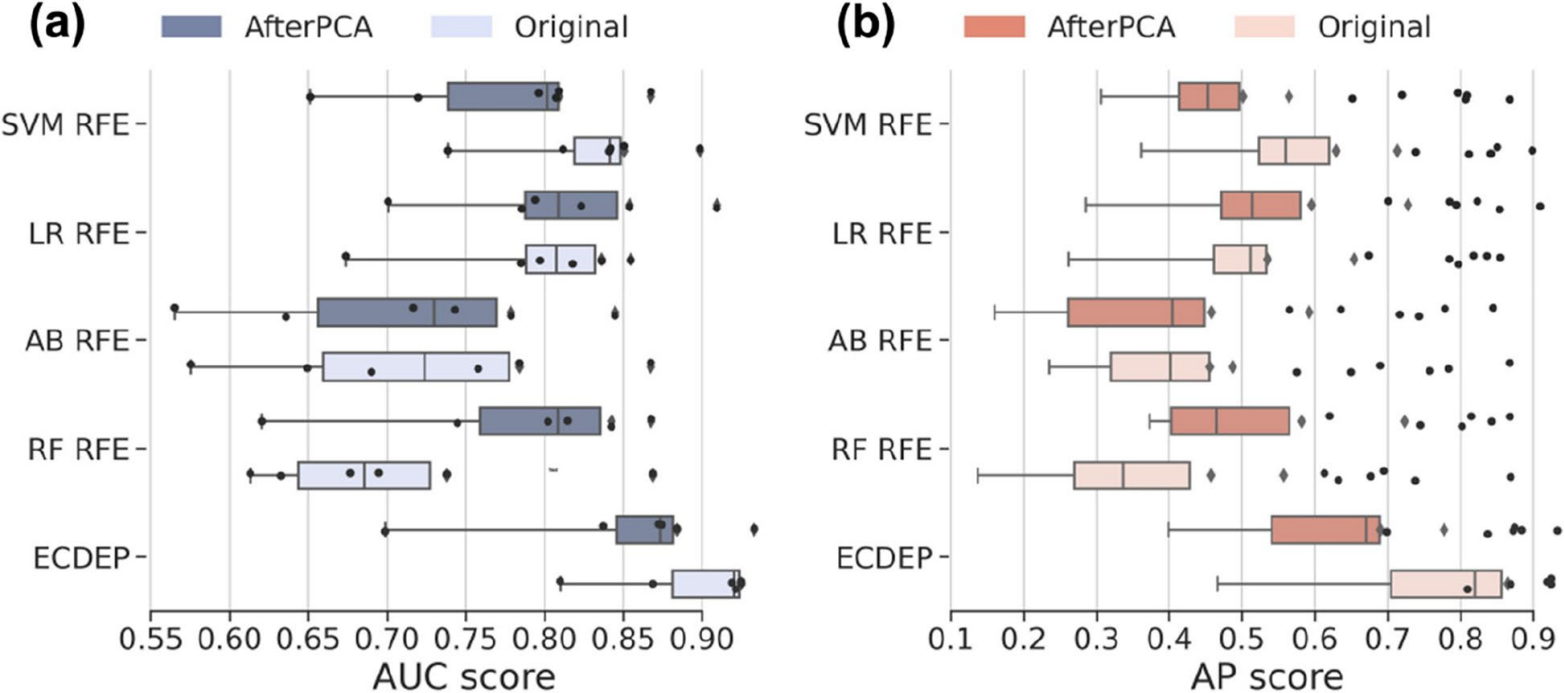
Comparing AUC and AP Values for ECDEP and Other ML Methods with PCA-Based Feature Extraction. **a** AUC Values of ECDEP and Other Machine Learning Methods after PCA-Based Feature Extraction. **b** AP Values of ECDEP and Other Machine Learning Methods after PCA-Based Feature Extraction. SVM + RFE: Support Vector Machine with Recursive Feature Elimination; AB + RFE: AdaBoost with Recursive Feature Elimination; LR + RFE: Logistic Regression with Recursive Feature Elimination; RF + RFE: Random Forest with Recursive Feature Elimination

However, as illustrated in Fig. 18 the performance of the ECDEP model combined with PCA was less than satisfactory. This could be attributed to the loss of important information contained in the features that were omitted during PCA, resulting in a decrease in model performance. On the other hand, we also applied PCA to machine learning methods. Interestingly, we observed that the results improved only for AB + RFE and RF + RFE. This highlights the effectiveness of PCA in feature selection for specific machine learning algorithms, such as AB and RF, combined with RFE. These results further underscore the complex interplay between feature selection techniques, data dimensionality reduction, and their impact on model performance.

## Conclusions

In this article, we propose ECDEP, a deep learning model grounded on evolutionary community discovery. ECDEP leverages PPI networks, gene expression profiles, and subcellular localization data as inputs to tackle the underutilization of deep learning in network dynamics and the limited contribution of gene expression data in classification tasks.

The model begins by incorporating gene expression data containing temporal information and identifies outlier gene expression levels at each time step using the 3-Sigma criterion. If the expression of nodes in the static network is abnormal at a specific time, the corresponding edges will be removed. After establishing a dynamic network, we calculate the interaction streaming sources by tracking newly generated and disappeared edges at each moment. Evolutionary community discovery techniques are applied to extract overlapping communities from the dynamic network, and SVM-RFE is employed to optimize the subset of community features. Finally, the selected community features are combined with subcellular localization data and fed into the classification module for prediction. Gene expression data in ECDEP contributes temporal information but doesn't directly influence the final decision-making process. This approach enhances the predictive capability of gene expression data.

The experiments in this article encompass three *S. cerevisiae* databases and datasets from three additional species. We compare ECDEP with ten centrality-based network topology methods, four shallow machine learning methods combined with RFE, and two deep learning methods incorporating multiple biological data sources. F-measure, AUC score, and AP score are used as primary evaluation metrics due to the imbalanced learning problem. Results indicate that while ECDEP performs slightly worse than MBIEP on the *S. cerevisiae* (BioGRID) dataset, it significantly outperforms other comparison methods on the *S. cerevisiae* (Krogan), *S. cerevisiae* (DIP), *H. sapiens*, *M. musculus*, and *C. elegans* datasets. Notably, on the *H. sapiens* dataset, ECDEP achieves an AP value 0.21 higher than MBIEP. Subsequently, we analyzed the contributions of the two biological inputs in the ECDEP model to prediction outcomes on various datasets. On the *S. cerevisiae* (Krogan) and *M. musculus* datasets, community contributions reached 0.46 and 0.57, respectively, highlighting ECDEP's ability to enhance the role of gene expression data. ECDEP solely relies on temporal information from gene expression data without restrictions on input, as gene expression data isn't directly utilized as a sample feature, thus improving the model's generalization capability.

However, the ECDEP model exhibits decreased performance on *M. musculus* and *C. elegans* datasets. Several factors contributing to this issue have been considered, including the selection of gene expression data, which is relevant to the model. Some gene expression data feature a time course, but their experimental environment and design, which have specific purposes, may introduce bias in subsequent functional community module detection. These challenges will be addressed in our future work.

### Supplementary Information

**Additional file 1: Figure S1.** Dynamic PPI Networks. **Figure S2.** Comparison with Centrality Methods. **Figure S3.** AUC and RP curves of ECDEP compared on *S. cerevisiae* (BioGRID) dataset. **Figure S4.** AUC and RP curves of ECDEP compared on *S. cerevisiae* (Krogan) dataset. **Figure S5.** AUC and RP curves of ECDEP compared on* M. musculus* dataset. **Figure S6.** AUC and RP curves of ECDEP compared on *C. elegans* dataset. **Figure S7.** AUC and RP curves of ECDEP compared on *S. cerevisiae* (DIP) dataset. **Figure S8.** Comparison with machine learning and deep learning methods. **Figure S9.** Ablation study of features in ECDEP across six datasets evaluated with AUC score. **Figure S10.** Process of detaching each snapshot. **Figure S11.** Evaluate the results of detaching each snapshot with F1, AUC, and AP scores. **Figure S12.** Comparison of information from static network and dynamic network. **Figure S13.** Generate the intersection set of ECDEP and EP-EDL methods. **Figure S14.** Comparison of ECDEP with RNN-based methods. **Figure S15.** Compare ECDEP with canonical Graph Convolutional Network (GCN). **Table S1.** Version and sources of databases. **Table S2.** Download links of methods for comparison. **Table S3.** Process of essential proteins for different species. **Table S4.** Process of gene expression profiles. **Table S5.** PPI network details for different species and datasets. **Table S6.** Environment, package, and version requirements. **Table S7.** Hyperparameter settings of ECDEP model. **Table S8.** Experiment on different selections of *M. musculus* essential protein.

## Data Availability

The code and data supporting the conclusions of this article are openly accessible in the GitHub Repository at https://github.com/LionKingAHAU/ECDEP. For additional figures and detailed information on the dataset employed in this paper, please refer to the Supplementary Materials.

## References

[1] Elizabeth A, Daniel D, Anna A (1999). Functional Characterization of the S. cerevisiae genome by gene deletion and parallel analysis. Science.

[2] Kamath R, Fraser A, Dong Y (2003). Systematic functional analysis of the Caenorhabditis elegans genome using RNAi. Nature.

[3] Furney SJ, Albà MM, López-Bigas N (2006). Differences in the evolutionary history of disease genes affected by dominant or recessive mutations. BMC Genomics.

[4] Steinmetz L, Scharfe C, Deutschbauer A (2002). Systematic screen for human disease genes in yeast. Nat Genet.

[5] Giaever G, Chu A, Ni L (2002). Functional profiling of the Saccharomyces cerevisiae genome. Nature.

[6] Cullen LM, Arndt GM (2005). Genome-wide screening for gene function using RNAi in mammalian cells. Immunol Cell Biol.

[7] Larry AG, Elizabeth R, Michael AJ (2007). A comprehensive transposon mutant library of Francisella novicida, a bioweapon surrogate. Proc Natl Acad Sci.

[8] Takashi I, Tomoko C, Ritsuko O, Mikio Y, Masahira H, Yoshiyuki S (2001). A comprehensive two-hybrid analysis to explore the yeast protein interactome. Proc Natl Acad Sci.

[9] Puig O, Caspary F, Rigaut G, Rutz B, Bouveret E, Bragado-Nilsson E (2001). The Tandem Affinity Purification (TAP) Method: A General Procedure of Protein Complex Purification. Methods..

[10] Ramsay G (1998). DNA chips: State-of-the art. Nat Biotechnol.

[11] Jeong H, Mason SP, Barabási AL, Oltvai ZN (2001). Lethality and centrality in protein networks. Nature.

[12] Wang H, Li M, Wang J, Pan Y. A New Method for Identifying Essential Proteins Based on Edge Clustering Coefficient. In: Chen J, Wang J, Zelikovsky A, editors. Bioinformatics Research and Applications. ISBRA 2011. Lecture Notes in Computer Science(), vol 6674. Berlin, Heidelberg: Springer; 2011. 10.1007/978-3-642-21260-4_12.

[13] Li M, Wang J, Chen X (2011). A local average connectivity-based method for identifying essential proteins from the network level. Comput Biol Chem.

[14] Qi Y, Luo J (2015). Prediction of essential proteins based on local interaction density. IEEE/ACM Trans Comput Biol Bioinf.

[15] Li M, Zhang H, Wang JX (2012). A new essential protein discovery method based on the integration of protein-protein interaction and gene expression data. BMC Syst Biol.

[16] Watts DJ, Strogatz SH (1998). Collective dynamics of 'small-world' networks. Nature.

[17] Tang X, Wang J, Zhong J (2013). Predicting essential proteins based on weighted degree centrality. IEEE/ACM Trans Comput Biol Bioinf.

[18] Zhang X, Xu J, Xiao W (2013). A new method for the discovery of essential proteins. PLoS One.

[19] Zhao B, Zhao Y, Zhang X (2019). An iteration method for identifying yeast essential proteins from heterogeneous network. BMC Bioinformatics.

[20] Li S, Zhang Z, Li X, Tan Y, Wang L, Chen Z (2021). An iteration model for identifying essential proteins by combining comprehensive PPI network with biological information. BMC Bioinformatics.

[21] Meng Z, Kuang L, Chen Z, Zhang Z, Tan Y, Li X (2021). Method for essential protein prediction based on a novel weighted protein-domain interaction network. Front Genet.

[22] Xu W, Dong Y, Guan J (2022). Identifying essential proteins from protein–protein interaction networks based on influence maximization. BMC Bioinformatics.

[23] Zhong J, Tang C, Peng W, Xie M, Sun Y, Tang Q (2021). A novel essential protein identification method based on PPI networks and gene expression data. BMC Bioinformatics.

[24] Liu P, Liu C, Mao Y (2023). Identification of essential proteins based on edge features and the fusion of multiple-source biological information. BMC Bioinformatics.

[25] Zhong J, Wang J, Peng W, Zhang Z, Li M (2015). A feature selection method for prediction essential protein. Tsinghua Sci Technol.

[26] Zhong J, Sun Y, Peng W, Xie M, Yang J, Tang X (2018). XGBFEMF: An XGBoost-Based Framework for Essential Protein Prediction. IEEE Trans Nanobioscience.

[27] Zeng M, Li M, Wu FX, Li Y, Pan Y (2019). DeepEP: A deep learning framework for identifying essential proteins. BMC Bioinformatics.

[28] Grover A, Leskovec J (2016). Node2vec: Scalable feature learning for networks. Proc ACM SIGKDD Int Conf Knowl Discov Data Min.

[29] Zeng M, Li M, Fei Z, Wu F-X, Li Y, Pan Y (2021). A Deep Learning Framework for Identifying Essential Proteins by Integrating Multiple Types of Biological Information. IEEE/ACM Trans Comput Biol Bioinforma.

[30] Yue Y, Ye C, Peng PY (2022). A deep learning framework for identifying essential proteins based on multiple biological information. BMC Bioinformatics.

[31] Lei X, Fang M, Guo L (2019). Protein complex detection based on flower pollination mechanism in multi-relation reconstructed dynamic protein networks. BMC Bioinformatics.

[32] Rossetti G, Pappalardo L, Pedreschi D (2017). Tiles: an online algorithm for community discovery in dynamic social networks. Mach Learn.

[33] Maliackal PJ, Amy B, Ingber D (2005). High-Betweenness Proteins in the Yeast Protein Interaction Network. J Biomed Biotechnol.

[34] Wuchty S, Stadler PF (2003). Centers of complex networks. J Theor Biol.

[35] Estrada E (2006). Virtual identification of essential proteins within the protein interaction network of yeast. Proteomics.

[36] Hage P, Harary F (1995). Eccentricity and centrality in networks. Soc Networks.

[37] Lin CY, Chin CH, Wu HH (2008). Hubba: hub objects analyzer—a framework of interactome hubs identification for network biology. Nucleic Acids Res.

[38] Oughtred R, Rust J, Chang C (2021). The BioGRID database: A comprehensive biomedical resource of curated protein, genetic, and chemical interactions. Protein Sci.

[39] Krogan NJ, Cagney G, Yu H (2006). Global landscape of protein complexes in the yeast Saccharomyces cerevisiae. Nature.

[40] Xenarios I, Rice DW, Salwinski L (2000). DIP: the database of interacting proteins. Nucleic Acids Res.

[41] Luo H, Lin Y, Liu T (2021). DEG 15, an update of the Database of Essential Genes that includes built-in analysis tools. Nucleic Acids Res.

[42] Gurumayum S, Jiang P, Hao X (2021). OGEE v3: Online GEne Essentiality database with increased coverage of organisms and human cell lines. Nucleic Acids Res.

[43] Zhang X, Xiao W, Xiao W (2020). DeepHE: Accurately predicting human essential genes based on deep learning. PLoS Comput Biol.

[44] Binder JX, Pletscher-Frankild S, Tsafou K (2014). COMPARTMENTS: unification and visualization of protein subcellular localization evidence. Database.

[45] Tu BP, Kudlicki A, Rowicka M (2005). Logic of the yeast metabolic cycle: temporal compartmentalization of cellular processes. Science.

[46] Yin X, Luistro L, Zhong H (2013). RG7212 anti-TWEAK mAb inhibits tumor growth through inhibition of tumor cell proliferation and survival signaling and by enhancing the host antitumor immune response. Clin Cancer Res.

[47] Sene KH, Porter CJ, Palidwor G (2007). Gene function in early mouse embryonic stem cell differentiation. BMC Genomics.

[48] Hou L, Wang D, Chen D (2016). A systems approach to reverse engineer lifespan extension by dietary restriction. Cell Metab.

[49] Edgar R, Domrachev M, Lash AE (2002). Gene Expression Omnibus: NCBI gene expression and hybridization array data repository. Nucleic Acids Res.

[50] Wang J, Peng X, Li M (2013). Construction and application of dynamic protein interaction network based on time course gene expression data. Proteomics.

[51] Rossetti G, Cazabet R (2018). Community discovery in dynamic networks: a survey. ACM Comput Surv (CSUR).

[52] Guyon I, Weston J, Barnhill S (2002). Gene selection for cancer classification using support vector machines. Mach Learn.

[53] Sha W, Martins AM, Laubenbacher R (2013). The genome-wide early temporal response of Saccharomyces cerevisiae to oxidative stress induced by cumene hydroperoxide. PLoS One.

[54] Li Y, Zeng M, Wu Y, Li Y, Li M (2021). Accurate prediction of human essential proteins using ensemble deep learning. IEEE/ACM Trans Comput Biol Bioinf.

[55] Li G, Muller M, Thabet A, Ghanem B (2019). Deepgcns: Can gcns go as deep as cnns?. Proceedings of the IEEE/CVF international conference on computer vision.

[56] Zeng M, Li M, Fei Z (2019). A deep learning framework for identifying essential proteins by integrating multiple types of biological information[J]. IEEE/ACM Trans Comput Biol Bioinf.

[57] Dey R, Salem FM. "Gate-variants of Gated Recurrent Unit (GRU) neural networks," 2017 IEEE 60th International Midwest Symposium on Circuits and Systems (MWSCAS). Boston; 2017. p. 1597–600. 10.1109/MWSCAS.2017.8053243.

[58] Liu J, Yang Y, Lv S, et al. Attention-based BiGRU-CNN for Chinese question classification. J Ambient Intell Human Comput. 2019. 10.1007/s12652-019-01344-9.

[59] Shi X, Chen Z, Wang H, et al. Convolutional LSTM Network: a machine learning approach for precipitation nowcasting[C]//Proceedings of the 28th International Conference on Neural Information Processing Systems-Volume 1. 2015. p. 802–10.

[60] Siami-Namini S, Tavakoli N, Namin AS. "The Performance of LSTM and BiLSTM in Forecasting Time Series." 2019 IEEE International Conference on Big Data (Big Data). Los Angeles; 2019. p. 3285–92. 10.1109/BigData47090.2019.9005997.

