# Supplementary material for: ECDEP: identifying essential proteins based on evolutionary community discovery and subcellular localization
Source: BMC Genomics. 2024 Jan 26;25:117. doi: 10.1186/s12864-024-10019-5 (PMC10821549; doi:10.1186/s12864-024-10019-5)
Supplement: Supplementary file 1 — Additional file 1: Figure S1. Dynamic PPI Networks. Figure S2. Comparison with Centrality Methods. Figure S3. AUC and RP curves of ECDEP compared on S. cerevisiae (BioGRID) dataset. Figure S4. AUC and RP curves of ECDEP compared on S. cerevisiae (Krogan) dataset. Figure S5. AUC and RP curves of ECDEP compared on M. musculus dataset. Figure S6. AUC and RP curves of ECDEP compared on C. elegans dataset. Figure S7. AUC and RP curves of ECDEP compared on S. cerevisiae (DIP) dataset. Figure S8. Comparison with machine learning and deep learning methods. Figure S9. Ablation study of features in ECDEP across six datasets evaluated with AUC score. Figure S10. Process of detaching each snapshot. Figure S11. Evaluate the results of detaching each snapshot with F1, AUC, and AP scores. Figure S12. Comparison of information from static network and dynamic network. Figure S13. Generate the intersection set of ECDEP and EP-EDL methods. Figure S14. Comparison of ECDEP with RNN-based methods. Figure S15. Compare ECDEP with canonical Graph Convolutional Network (GCN). Table S1. Version and sources of databases. Table S2. Download links of methods for comparison. Table S3. Process of essential proteins for different species. Table S4. Process of gene expression profiles. Table S5. PPI network details for different species and datasets. Table S6. Environment, package, and version requirements. Table S7. Hyperparameter settings of ECDEP model. Table S8. Experiment on different selections of M. musculus essential protein. [file 12864_2024_10019_MOESM1_ESM.docx]

**Supplementary Materials**

**ECDEP: Identifying essential proteins based on evolutionary community discovery and subcellular localization**

Chen Ye^1,2,+^, Qi Wu^1,2,+^, Shuxia Chen ^1,2^, Xuemei Zhang^1,2^, Wenwen Xu^1,2^, Yunzhi Wu^1,2^, Youhua Zhang^1,2^ and Yi Yue^1,2,*^

^1^ School of Information and Artificial Intelligence, Anhui Agricultural University, Hefei, Anhui 230036, China.

^2^ Anhui Beidou Precision Agriculture Information Engineering Research Center, Anhui Agricultural University, Hefei 230036, China.

**^+^** These authors contributed equally to this work.

**^*^** Corresponding author: yyyue@ahau.edu.cn

**Figure S1.** Dynamic PPI Networks

The process of PPI networks of different species over time and the network status at each moment: the number of existing interactions, the number of existing nodes, the number of interactions that disappeared at that moment, and the number of new interactions that emerged at that moment. a) Dynamic PPI network of three different *S. cerevisiae* databases (constructed through GSE3431); b) *H. sapiens* dynamic PPI network (constructed through GSE41828); c) *M. musculus* dynamic PPI network (constructed through GSE3231); d) *C. elegans* dynamic PPI network (constructed through GSE77110).


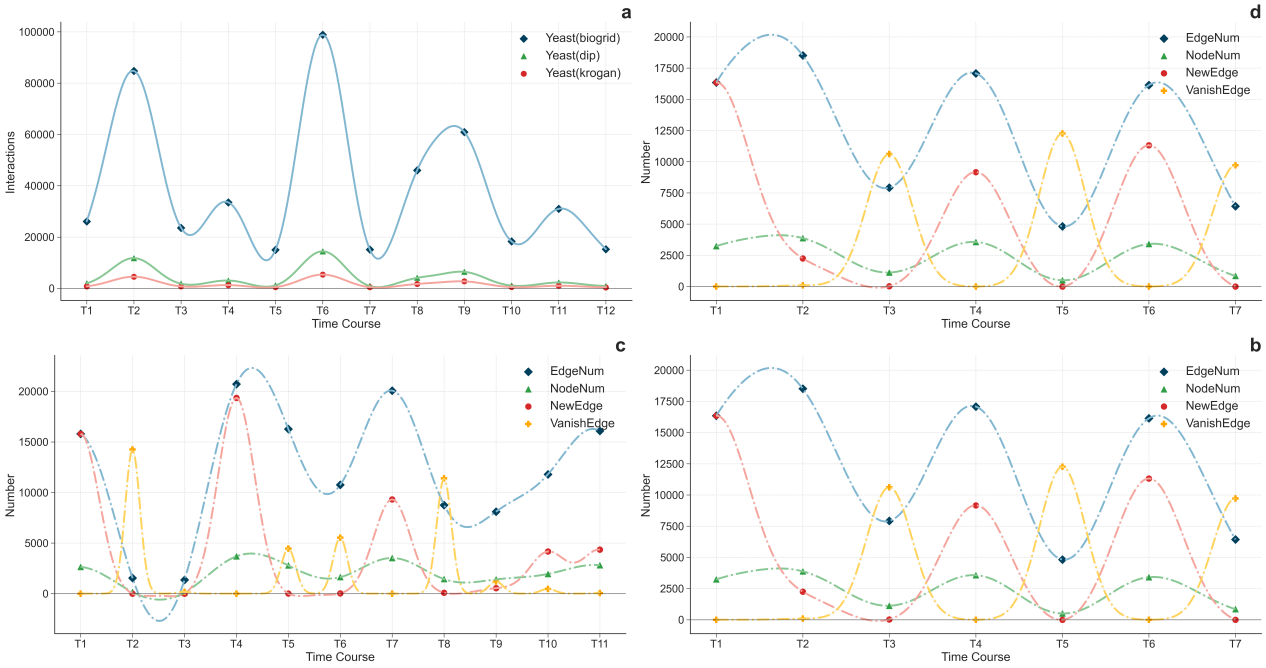


**Figure S2.** Comparison with Centrality Methods

The ECDEP model was compared with ten different centrality methods on six different datasets/species for other metrics. a) In terms of accuracy indicators, ECDEP outperforms all comparison methods on all datasets; b) ECDEP outperforms all comparison methods on all datasets in terms of precision indicators; c) ECDEP outperformed all comparison methods in terms of Recall metrics across all datasets.


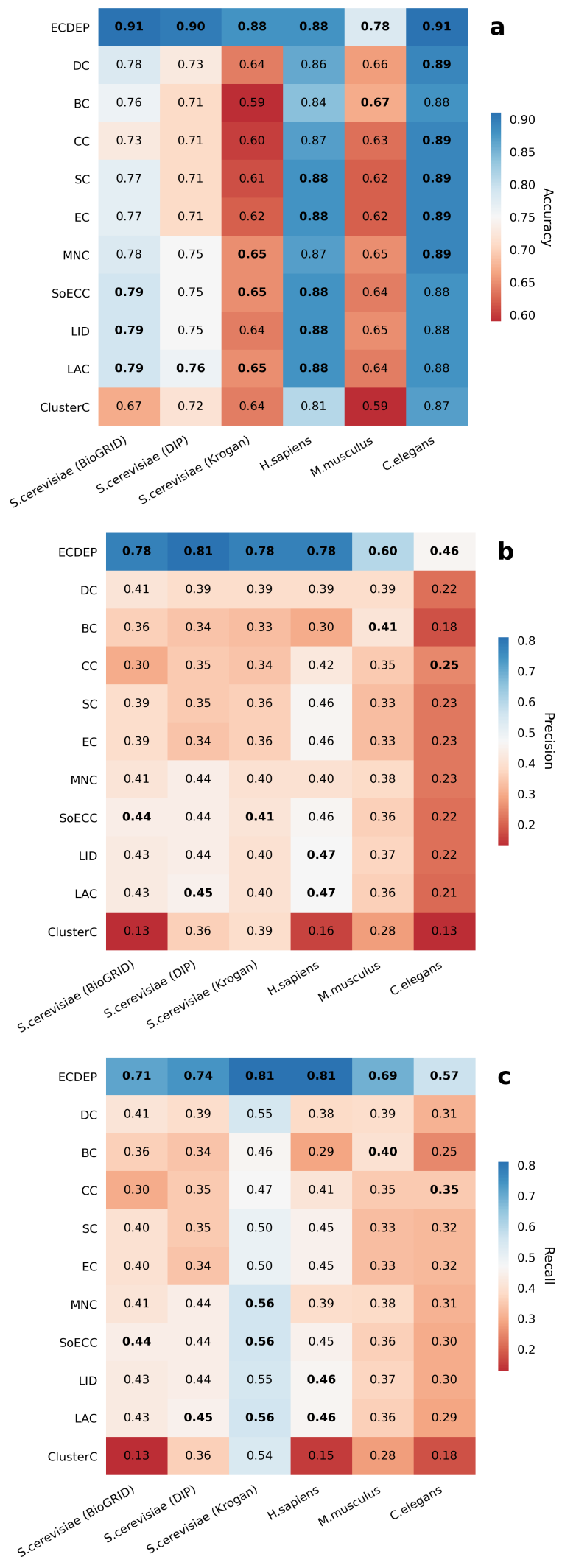


**Figure S3.** AUC and RP curves of ECDEP compared on *S. cerevisiae* (BioGRID) dataset

ECDEP achieves an AP value identical to that of MBIEP while displaying a slight reduction of 0.01 in the AUC value compared to MBIEP. Notably, ECDEP outperforms DeepEP with an AUC value that is 0.12 higher and an AP value that is 0.42 higher. Furthermore, our results indicate that ECDEP's performance surpasses that of all shallow machine learning models included in the comparison. It is important to highlight that these models extra utilize subcellular localization data as input, resulting in significantly improved predictive capabilities. This underscores the crucial role of subcellular localization information in enhancing the precision of essential protein prediction tasks.


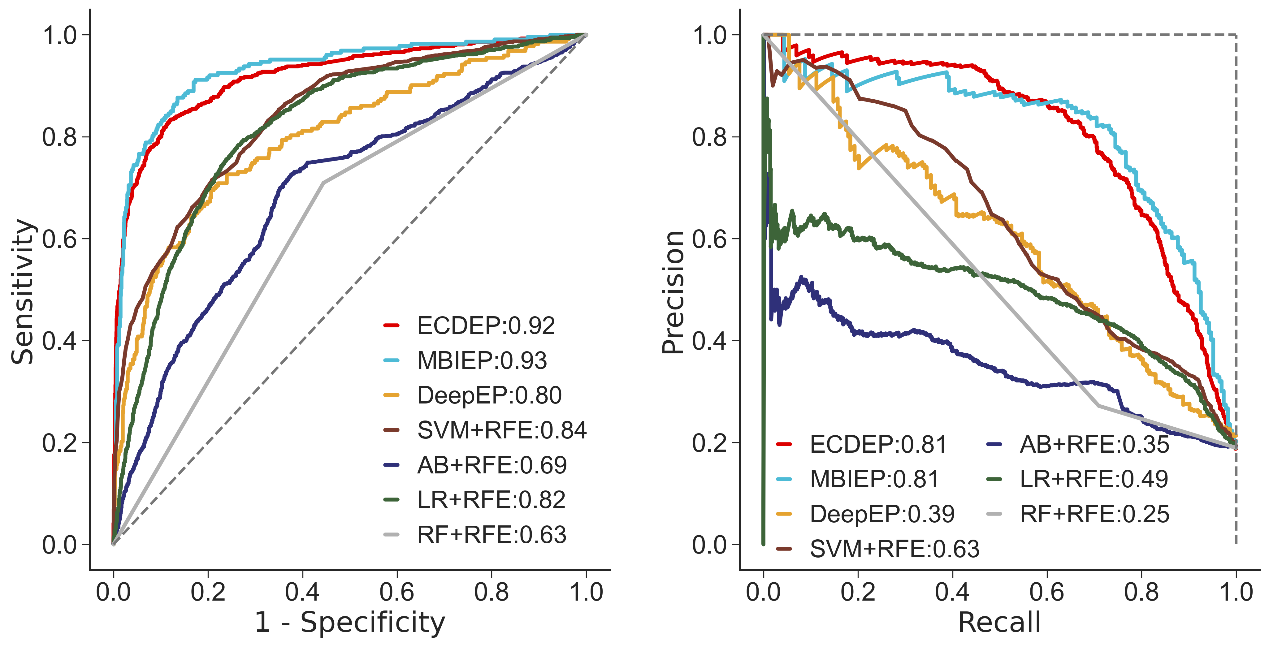


**Figure S4.** AUC and RP curves of ECDEP compared on *S. cerevisiae* (Krogan) dataset

ECDEP continues to outperform other models, with AUC and AP values that exceed those of MBIEP by 0.03 and 0.07, DeepEP by 0.15 and 0.43, and the leading SVM-RFE by 0.07 and 0.15, respectively. Additionally, compared to the BioGRID database, the majority of algorithms demonstrated improved performance. The Krogan dataset stands out due to its unique characteristics, all interaction edges are experimentally confirmed, resulting in lower network noise and a higher proportion of essential proteins. This reduces the challenges associated with imbalanced learning in the dataset.


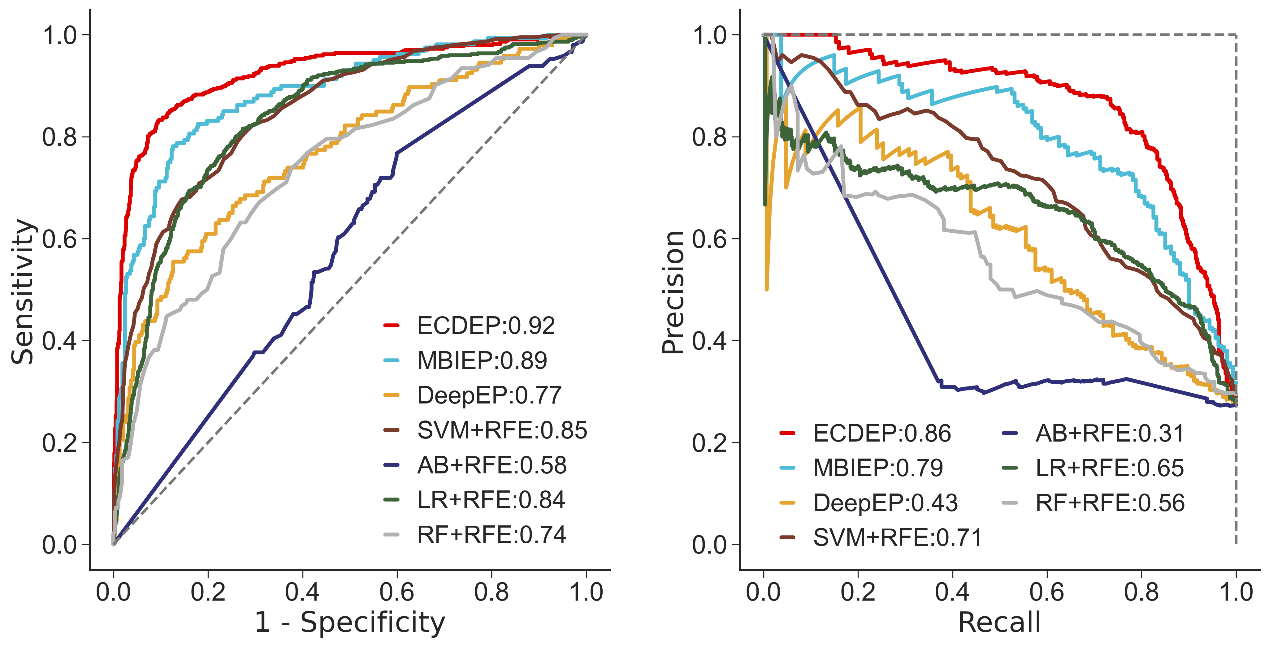


**Figure S5.** AUC and RP curves of ECDEP compared on *M. musculus* dataset

Our interest lies in understanding the variations in performance on the *M. musculus* dataset, given the high biological similarity between *M. musculus* and *H. sapiens*, including numerous homologous genes. We initially explored different labeling approaches, including applying the DEG database alone, employing the OEGG dataset alone, selecting the intersection of both databases, and choosing data from both homologous genes and essential proteins in both *H. sapiens* and *M. musculus* species. Unfortunately, the experimental results fail to meet our expectations. Therefore, while ECDEP clearly outperforms all comparative methods on the *M. musculus* dataset, there remains an opportunity for further refinement and enhancement.


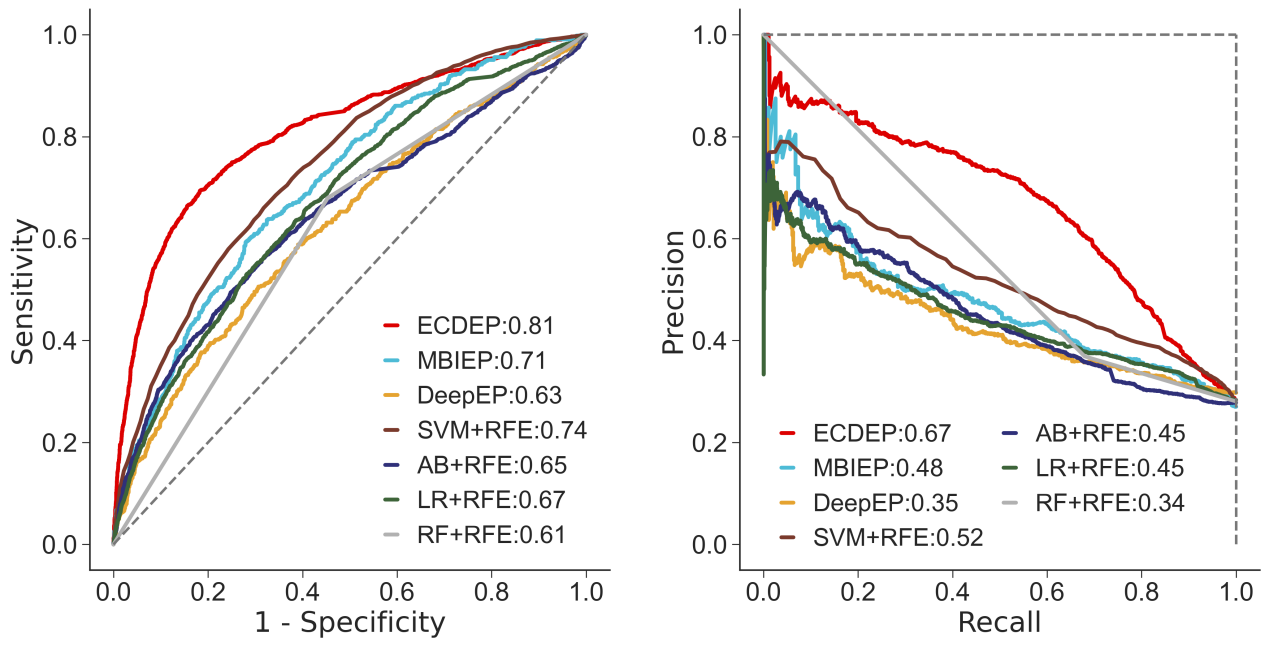


**Figure S6.** AUC and RP curves of ECDEP compared on *C. elegans* dataset

All methods experience a significant decline in AP values on the *C. elegans* dataset, which holds the lowest proportion of essential proteins among all datasets, exacerbating its imbalanced learning challenge. Upon comparison, we find that despite its lower proportion of essential proteins, the *C. elegans* dataset yields a relatively higher AUC values for the algorithms. This phenomenon can be attributed to the dataset's elevated ratio of negative samples, enabling the models to correctly identify more negative instances. However, the models struggle to accurately detect positive samples, resulting in high AUC but low AP scores. Similarly, while ECDEP maintains its superiority over all comparison algorithms on the *C. elegans* dataset, its performance has markedly declined, which can be ascribed to the dataset's limited number of positive samples and the inherent differences between species.


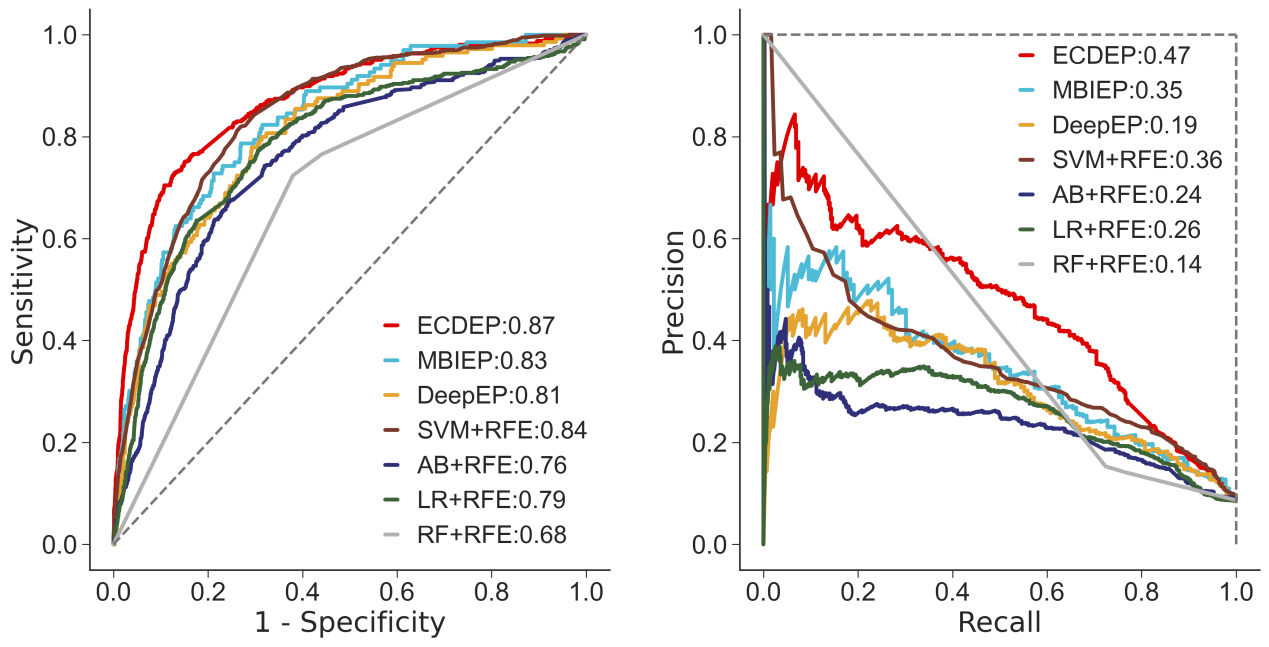


**Figure S7.** AUC and RP curves of ECDEP compared on *S. cerevisiae* (DIP) dataset

We further assess the performance of the ECDEP model on two additional *S. cerevisiae* databases. On the *S. cerevisiae* (DIP) dataset, ECDEP exhibits superior performance compared to other methods. Specifically, it achieved AUC and AP values that are 0.04 and 0.10 higher than MBIEP, 0.16 and 0.43 higher than DeepEP, and 0.11 and 0.24 higher than the best-performing SVM-RFE among shallow machine learning methods. Notably, compared to the BioGRID dataset, most algorithms demonstrate a modest improvement in performance. It's worth mentioning that the BioGRID dataset possesses a larger network scale and density, presenting a challenge in effectively leveraging its rich and timely interaction data while mitigating noise.


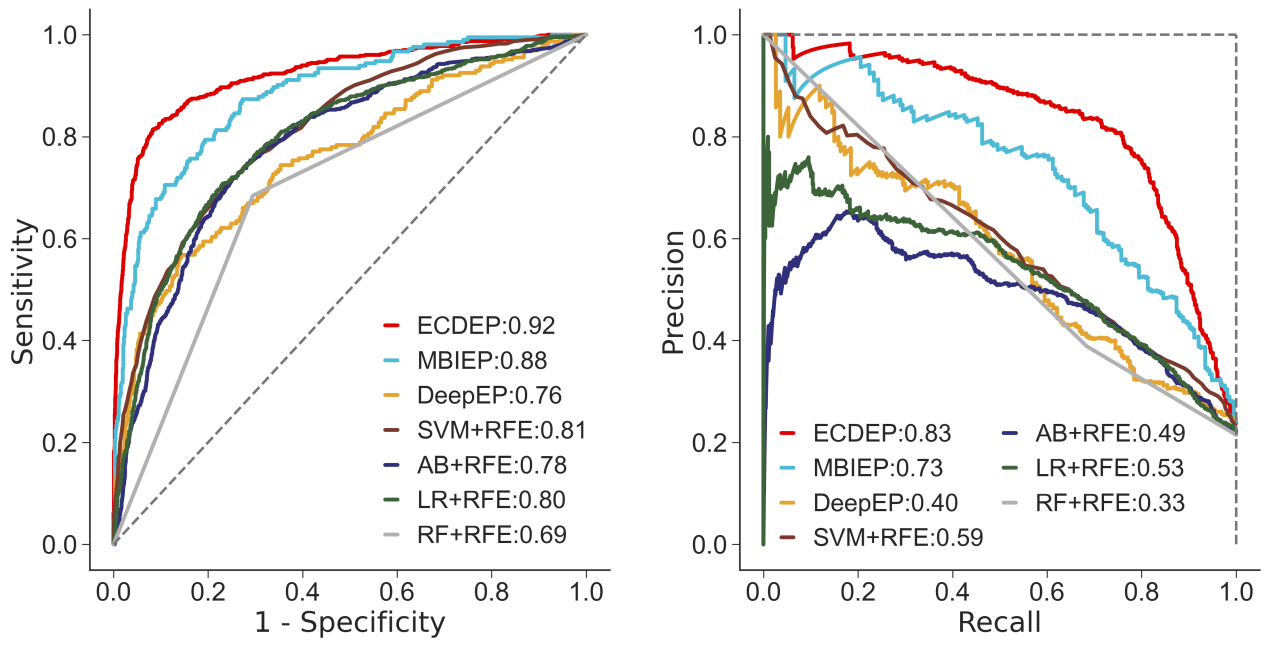


**Figure S8.** Comparison with machine learning and deep learning methods

**
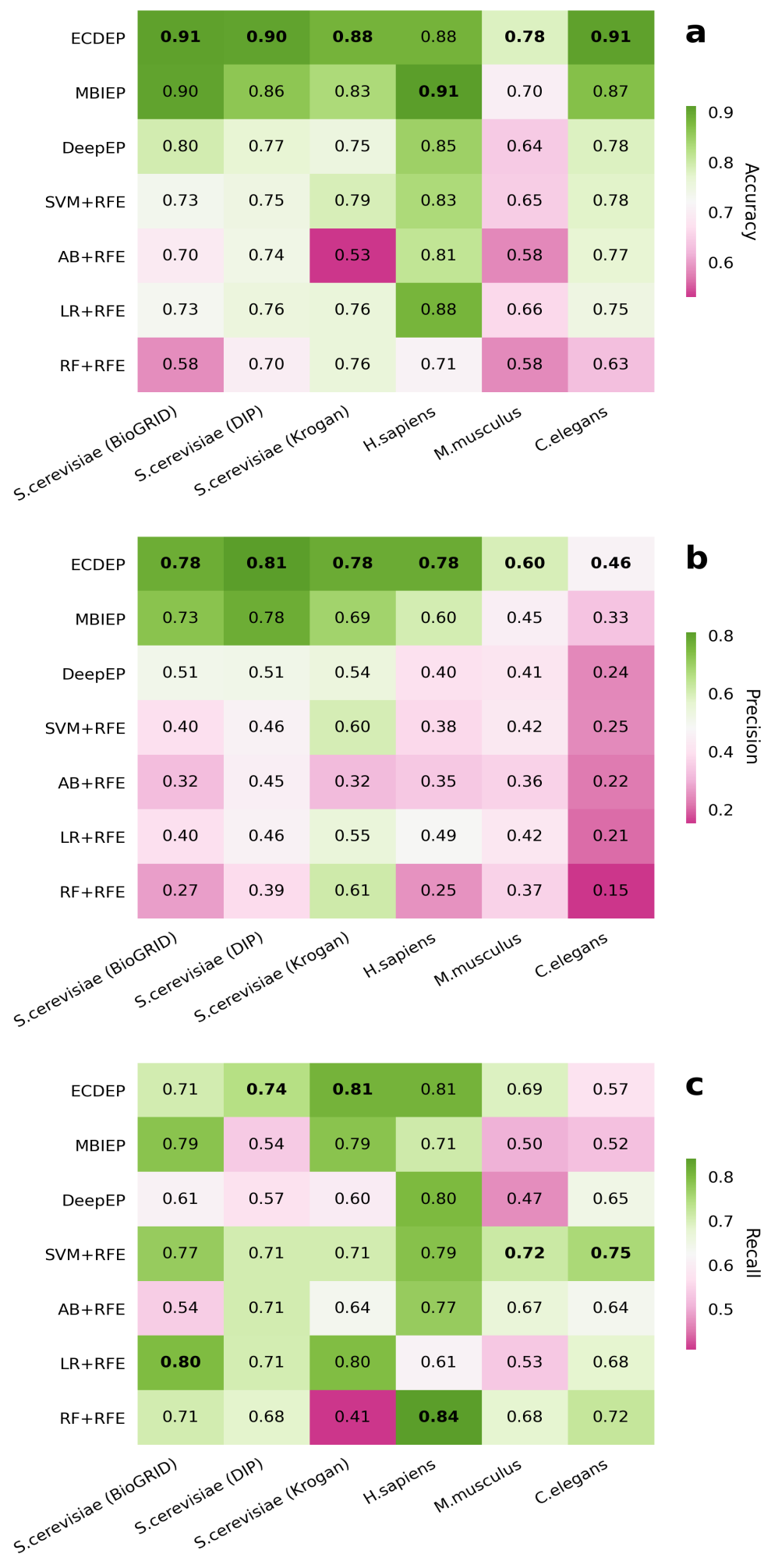
**

**Figure S9.** Ablation study of features in ECDEP across six datasets evaluated with AUC score

(a)~(f) exhibit different species and datasets. COM: community feature; SUB: subcellular localization feature; COM+SUB: community feature combined with subcellular localization feature.

**
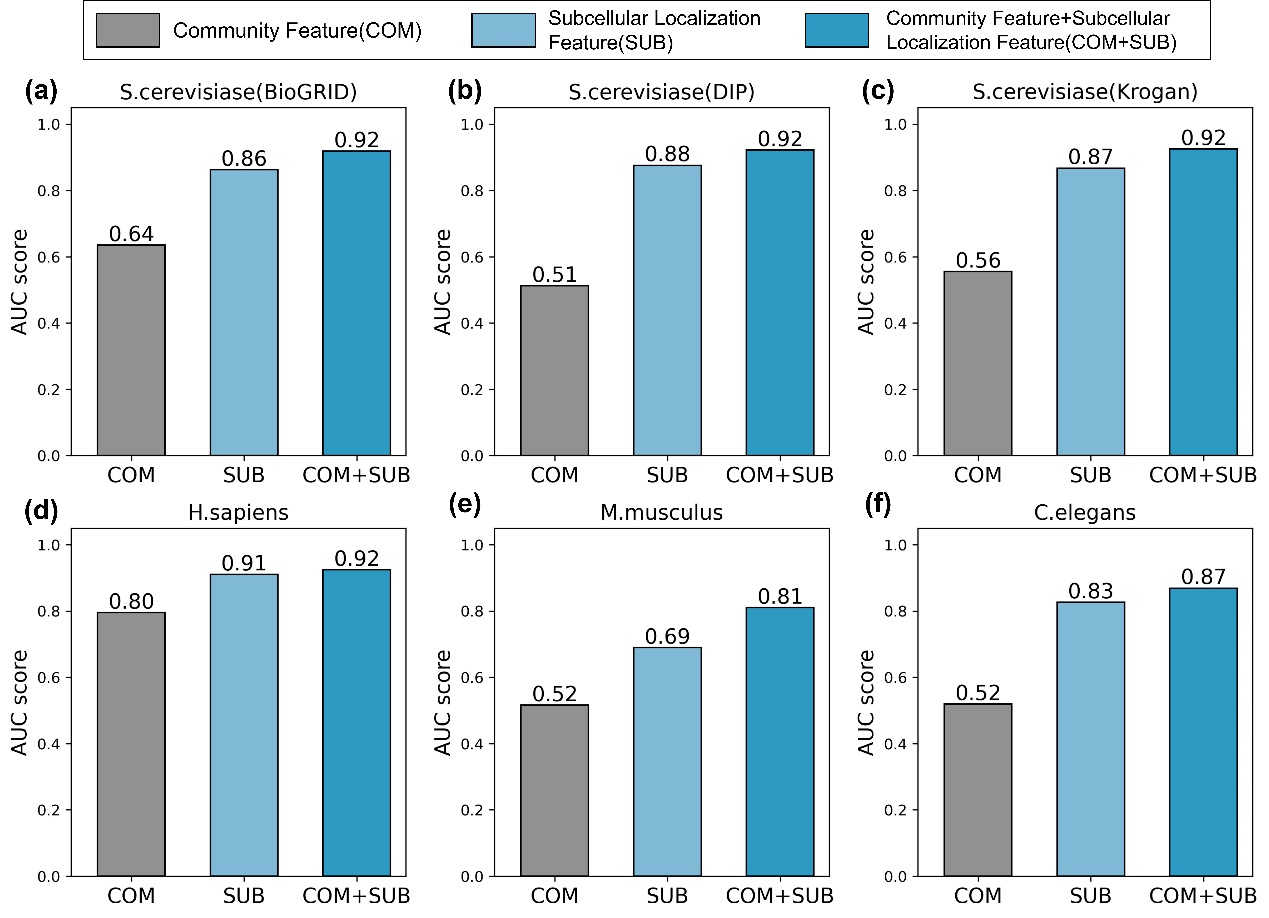
**

**Figure S10.** Process of detaching each snapshot

With Ti represents each frame of dynamic Protein-Protein Interaction network, we detach every snapshot to observe its impact on results.


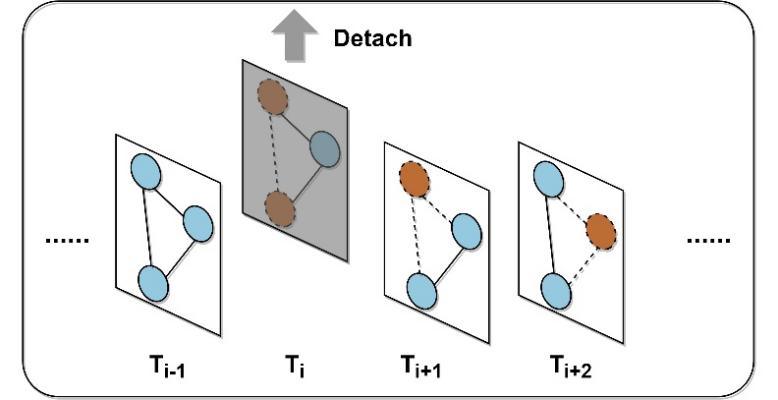


**Figure S11.** Evaluate the results of detaching each snapshot with F1, AUC, and AP scores

Red dashed line in each subgraph denotes the corresponding value of ECDEP. Each point on the X-axis represents an extraction operation of snapshot Ti, while the corresponding value on the Y-axis represents the evaluation result of that operation.


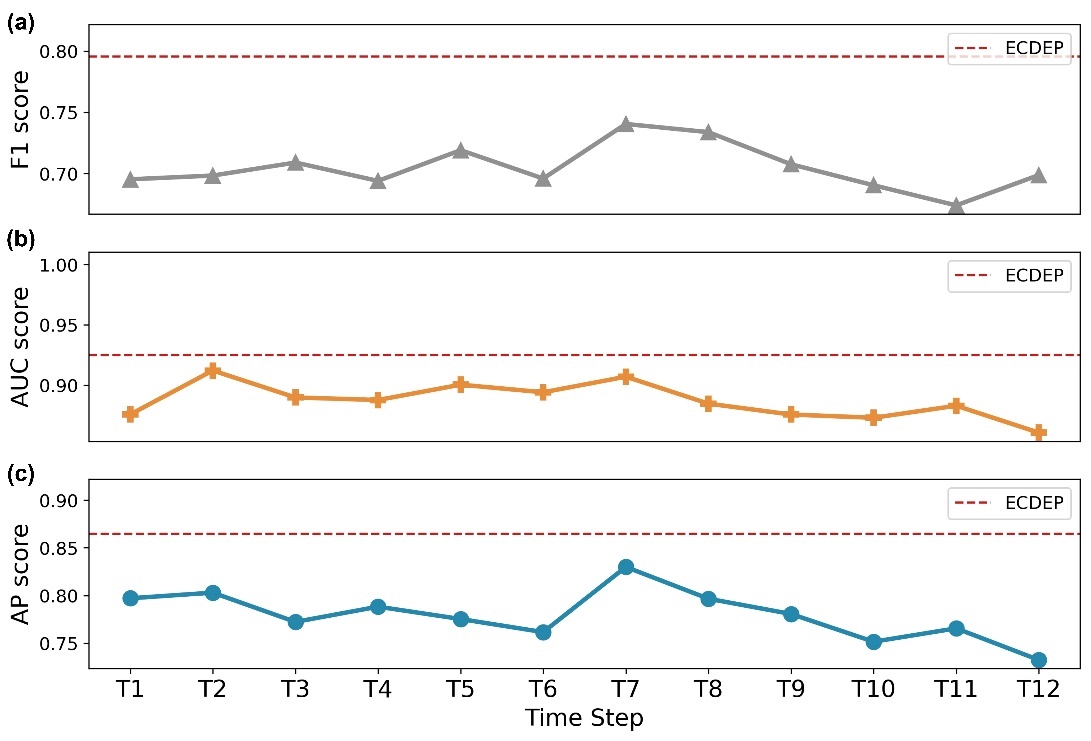


**Figure S12.** Comparison of information from static network and dynamic network.

(a)~(c) represent evaluation with F1, AUC and AP scores, respectively. static_: Substitute the community feature in our model with network embedding feature from static protein-protein interaction network. dynamic_: Process of dynamic network in ECDEP. S.C(B): Saccharomyces cerevisiae (BioGRID); S.C(D): Saccharomyces cerevisiae (DIP); S.C(K): Saccharomyces cerevisiae (Krogan); H.S: Homo sapiens; M.M: Mus musculus; C.E: Caenorhabditis elegans.


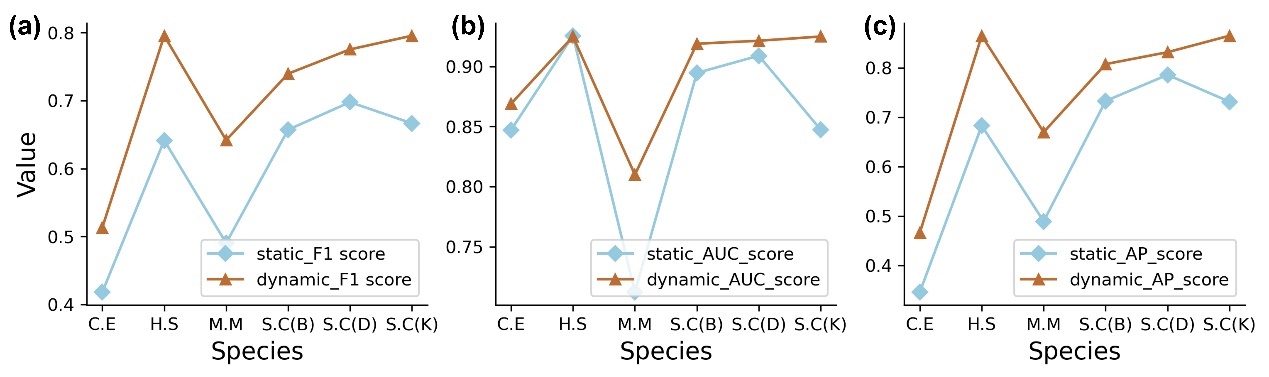
**Figure S13.** Generate the intersection set of ECDEP and EP-EDL methods

In Preprocess section, the unmapped proteins are converted with Uniprot ID Mapping. In Build Intersection Dataset section, the new protein dataset is built on the intersection of datasets from two methods.


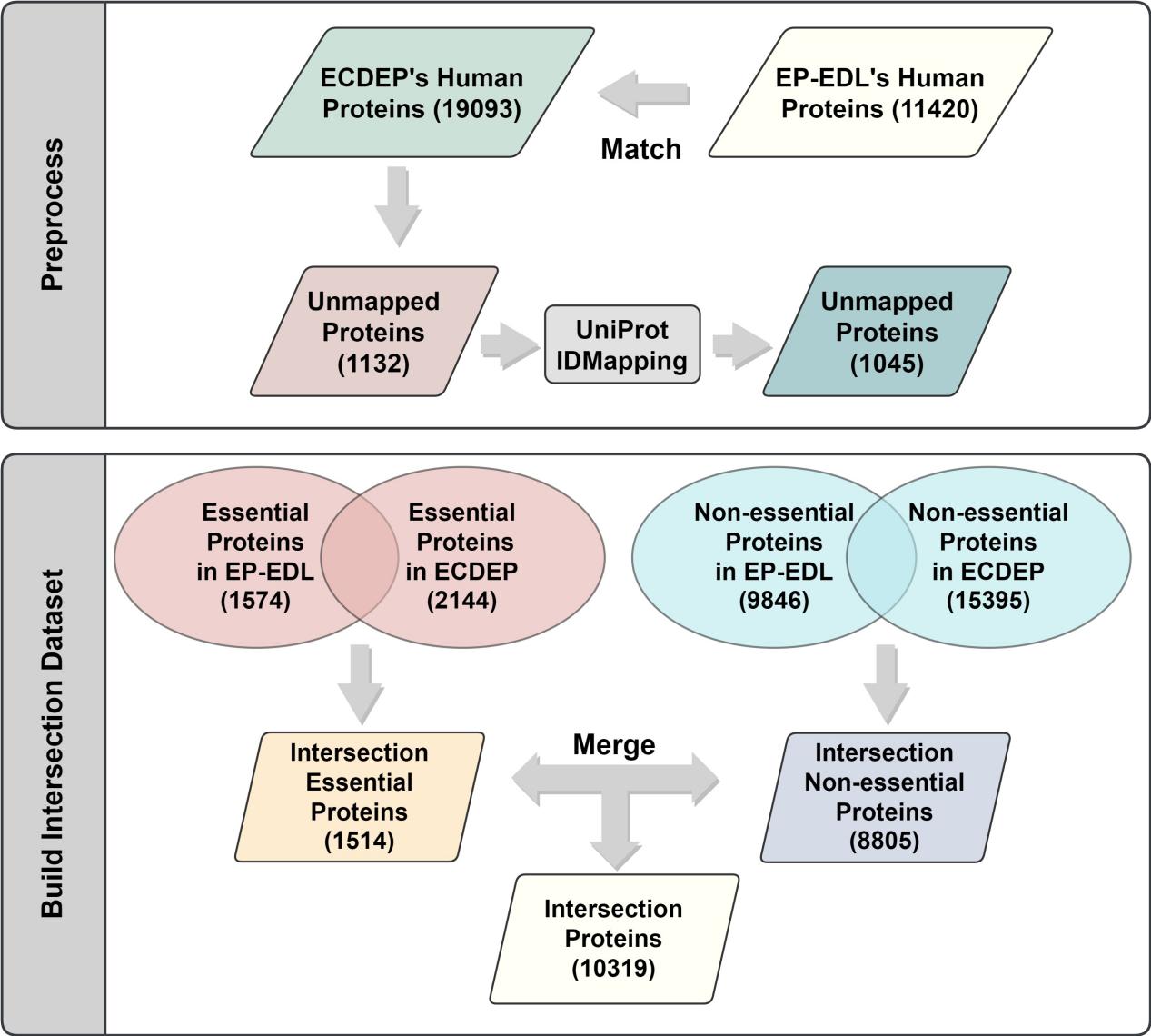


**Figure S14.** Comparison of ECDEP with RNN-based methods

BiGRU: Bidirectional Gated recurrent unit; GRU: Gated recurrent unit; BiLSTM: Bidirectional Long short-term memory, LSTM: Long short-term memory.

*
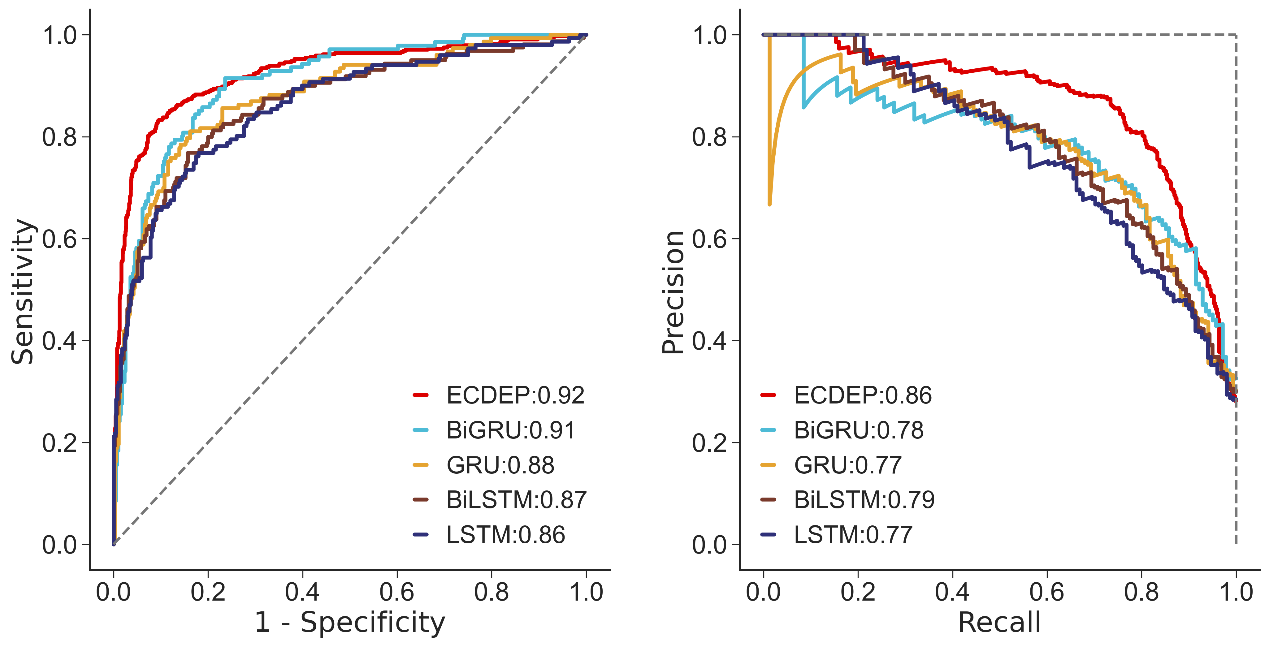
*

**Figure S15.** Compare ECDEP with canonical Graph Convolutional Network (GCN)

c_: community feature; s_: subcellular localization feature’ s_256_: 256-dimension subcellular localization feature with SVM-RFE. s+c_: subcellular localization feature and community feature; 2GCN: implemented with 2-layer GCN networks; 3GCN: implemented with 3-layer GCN networks.


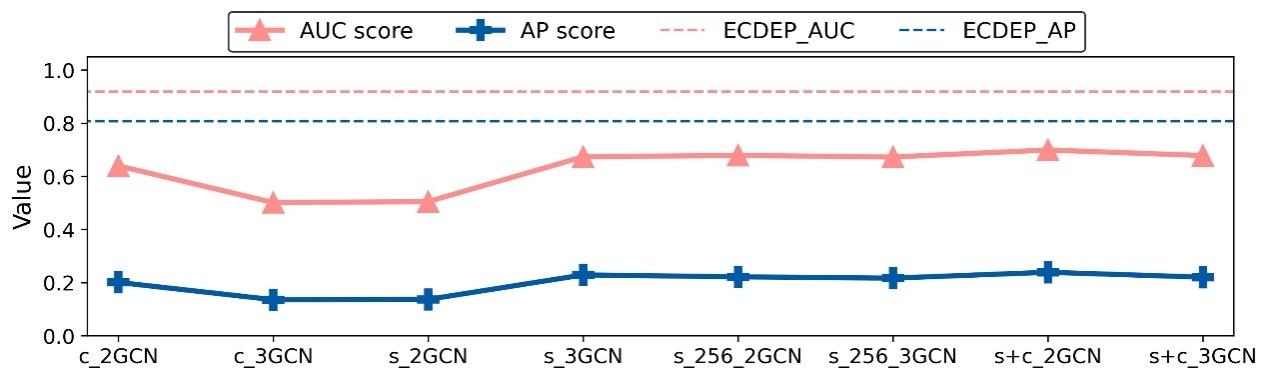
**Table S1.** Version and sources of databases

| **Data Type** | **Database** | **Download Link** | **Version** |
| --- | --- | --- | --- |
| Protein-Protein Interaction Network | BioGRID | https://downloads.thebiogrid.org/BioGRID | 4.4.200 |
|  | BioGRID | https://downloads.thebiogrid.org/BioGRID | 4.4.200 |
|  | Krogan dataset | https://www.yeastgenome.org/reference/S000114782#file | nan |
| Gene Expression Profiles | Gene Expression Omnibus | https://www.ncbi.nlm.nih.gov/geo/ | nan |
| Subcellular Localization | COMPARTMENTS database | https://compartments.jensenlab.org/Downloads | 20220630 |
| Essential Proteins | DEG database | http://origin.tubic.org/deg/public/index.php | 20200901 |
|  | OGEE database | https://v3.ogee.info/#/home | 20221007 |

**Table S2.** Download links of methods for comparison

| **Method** | **Link** | **Type** |
| --- | --- | --- |
| DC | https://networkx.org/documentation/stable/reference/algorithms/centrality.html | Centrality Methods |
| BC |  |  |
| CC |  |  |
| EC |  |  |
| SC |  |  |
| ClusterC | https://www.nature.com/articles/30918 |  |
| MNC | https://academic.oup.com/nar/article/36/suppl_2/W438/2506254 |  |
| SoECC | https://ieeexplore.ieee.org/document/6081844 |  |
| LAC | https://www.sciencedirect.com/science/article/abs/pii/S1476927111000296 |  |
| LID | https://ieeexplore.ieee.org/document/7360166 |  |
| SVM+RFE | https://scikit-learn.org/stable/ | Machine Learning Methods |
| RF+RFE |  |  |
| AB+RFE |  |  |
| LR+RFE |  |  |
| DeepEP | https://github.com/CSUBioGroup/DeepEP | Deep Learning Methods |
| MBIEP | https://github.com/LionKingAHAU/MBIEP |  |

**DC:** Degree Centrality; **BC:** Betweenness Centrality; **CC:** Closeness Centrality; **SC:** Subgraph Centrality; **EC:** Eigenvector Centrality; **MNC:** Maximum Neighborhood Component Centrality; **SoECC:** Sum of Edge Cluster Coefficient; **LID:** Local Interaction Density; **LAC:** Local Average Connectivity; **ClusterC:** Cluster Coefficient. **SVM+RFE:** Support Vector Machine with Recursive Feature Elimination; **AB+RFE:** AdaBoost with Recursive Feature Elimination; **LR+RFE:** Logistic Regression with Recursive Feature Elimination; **RF+RFE:** Random Forest with Recursive Feature Elimination.

**Table S3.** Process of essential proteins for different species

| **Species** | **Database** | **Num** | **Final Result** | **Proportion (%)** |
| --- | --- | --- | --- | --- |
| *S. cerevisiae* | OGEE | 946 | 1132 | 18.90 |
|  | DEG | 1109 |  |  |
| *M. musculus* | OGEE | 1578 | 2914 | 28.22 |
|  | DEG | 1908 |  |  |
| *C. elegans* | OGEE | 516 | 700 | 8.82 |
|  | DEG2002 | 294 |  |  |
|  | DEG2023 | 44 |  |  |
| *H. sapiens* | DEG | 20 DEG sets | 2161 | 11.32 |

**Proportion column** indicates the proportion of essential proteins in the PPI network.

**Table S4.** Process of gene expression profiles

| **Species** | **Accession** | **Description** | **Time Course** | **Our process** |
| --- | --- | --- | --- | --- |
| *S. cerevisiae* | GSE3431 | Three consecutive metabolic cycles of yeast | 12 time intervals | Calculate the average of three cycles. |
| *H. sapiens* | GSE41828 | TWEAK-treated time course in U2OS cells. | 5 time intervals | Select TWEAK treated group and calculate the average of five replicate samples. |
| *M. musculus* | GSE3231 | V6.5 Embryonic Stem Cell and Embryoid Body Time Course. | 11 time intervals | Select embryoid body group and calculate the average expression level of three replicate samples. |
| *C. elegans* | GSE77110 | C. elegans time course study on dietary restriction and aging. | 6 time intervals | Select intermittent fasting group. |

**Table S5.** PPI network details for different species and datasets

| **Database** | **Species** | **Proteins** | **Interactions** | **Average Degree** |
| --- | --- | --- | --- | --- |
| BioGRID | *H. sapiens* | 19093 | 537790 | 28.17 |
|  | *M. musculus* | 10170 | 55165 | 5.47 |
|  | *C. elegans* | 7934 | 32676 | 4.12 |
|  | *S. cerevisiae* | 5988 | 127581 | 21.31 |
| DIP |  | 4719 | 21913 | 4.64 |
| Krogan |  | 2674 | 7079 | 2.65 |

**Table S6.** Environment, package, and version requirements

| **Package/Environment** | **Version** | **Link** |
| --- | --- | --- |
| Python | >=3.8 | https://www.python.org/ |
| Numpy | >=1.19.4 | https://numpy.org/ |
| Pandas | >=1.0.5 | https://pandas.pydata.org/ |
| Tensorflow | >=2.4.0 | https://www.tensorflow.org/ |
| Sklearn | >=0.23.1 | https://scikit-learn.org/stable/ |
| Tiles | >=1.0.4 | https://github.com/GiulioRossetti/TILES |

**Table S7.** Hyperparameter settings of ECDEP model

| **Parameter Name** | **Range** | **Recommended Value** | |
| --- | --- | --- | --- |
| Epoch | 10, 20, 30, 40 | | 20 |
| Batch Size | 32, 64, 128, 512 | | 64 |
| n_features_to_select | 16, 32, 64, 128 | | 64 |
| Learning Rate | 0.1, 0.001, 0.002, 0.003, 0.0001 | | 0.001 |
| Optimizer | RMSprop, SGD, Adam, Adamax, Nadam, Adadelta | | Adamax |

**n_features_to_select** parameter refers to the number of features that SVM-RFE selects.

**Table S8.** Experiment on different selections of *M. musculus* essential protein

| **Metrics** | **DEG** | **OGEE** | **Union** | **Intersection** | **Conserved Genes** |
| --- | --- | --- | --- | --- | --- |
| Number | 1578 | 1908 | 2914 | 572 | 785 |
| F1 | 0.43 | 0.26 | **0.64** | 0.05 | 0.37 |
| ACC | 0.79 | 0.66 | 0.78 | **0.92** | 0.87 |
| PRE | 0.44 | 0.21 | **0.60** | 0.09 | 0.30 |
| REC | 0.42 | 0.35 | **0.69** | 0.03 | 0.50 |
| AUC | 0.67 | 0.54 | **0.81** | 0.58 | 0.83 |
| AP | 0.43 | 0.19 | **0.67** | 0.09 | 0.27 |

**F1**: F-measure; **ACC**: Accuracy; **PRE**: Precision; **REC**: Recall; **AUC**: AUC score; **AP**: AP score; **Union**: integrate two databases together and remove duplicates; **Intersection**: select essential genes presented in both databases; **Conserved Genes**: same essential genes that presented in *M. musculus* and *H. sapiens*.
